# Structural organization and energy storage in crosslinked actin assemblies

**DOI:** 10.1371/journal.pcbi.1006150

**Published:** 2018-05-29

**Authors:** Rui Ma, Julien Berro

**Affiliations:** 1 Department of Molecular Biophysics and Biochemistry, Yale University, New Haven, Connecticut, United States of America; 2 Nanobiology Institute, Yale University, West Haven, Connecticut, United States of America; 3 Department of Cell Biology, Yale University School of Medicine, New Haven, Connecticut, United States of America; University of Pittsburgh, UNITED STATES

## Abstract

During clathrin-mediated endocytosis in yeast cells, short actin filaments (< 200nm) and crosslinking protein fimbrin assemble to drive the internalization of the plasma membrane. However, the organization of the actin meshwork during endocytosis remains largely unknown. In addition, only a small fraction of the force necessary to elongate and pinch off vesicles can be accounted for by actin polymerization alone. In this paper, we used mathematical modeling to study the self-organization of rigid actin filaments in the presence of elastic crosslinkers in conditions relevant to endocytosis. We found that actin filaments condense into either a disordered meshwork or an ordered bundle depending on filament length and the mechanical and kinetic properties of the crosslinkers. Our simulations also demonstrated that these nanometer-scale actin structures can store a large amount of elastic energy within the crosslinkers (up to 10*k*_B_*T* per crosslinker). This conversion of binding energy into elastic energy is the consequence of geometric constraints created by the helical pitch of the actin filaments, which results in frustrated configurations of crosslinkers attached to filaments. We propose that this stored elastic energy can be used at a later time in the endocytic process. As a proof of principle, we presented a simple mechanism for sustained torque production by ordered detachment of crosslinkers from a pair of parallel filaments.

## Author summary

In many cellular processes that involve the deformation of membranes or the movement of vesicles and organelles, the energy from biochemical reactions is converted into forces. The biological filaments called actin are one of the major force producing machineries of the cell. It is commonly believed that the elongation of these filaments at their tip is the only way actin filaments can exert force. However, the amount of force produced by this mechanism can only account for a small fraction of the force in key cellular processes, such as clathrin-mediated endocytosis. In this paper, we demonstrate that connecting actin filaments with each other with flexible proteins called crosslinkers is a new way to transform biochemical energy into mechanical energy, and that this stored mechanical energy can be used to rotate filaments in a sustained direction. This mechanism of chemical energy conversion into mechanical work is a new paradigm for understanding how the actin filaments can produce forces without considering polymerization or molecular motors.

## Introduction

The cytoskeleton protein actin assembles into three major structures in yeast cells, including endocytic actin patches, actin cables, and the contractile ring [1, 2]. In actin cables and the contractile ring, formin-nucleated actin filaments are crosslinked into long bundles with a length on the order of microns [3–5]. Computational models of these actin structures typically treat actin filaments as semi-flexible polymers that are connected by rigid segments [6–9]. In contrast, the organization of the actin network in actin patches formed during clathrin-mediated endocytosis is drastically different from that in actin cables or the contractile ring. The length of filaments in actin patches is strongly limited by capping and severing proteins [10], and mathematical modeling predicted that the average length of filaments is less than 200 nm [11]. Filaments of this length scale can be considered as straight rods, because the persistence length of actin filaments is on the order of 10*μ*m [12–14], which allows them to sustain forces larger than 10pN without buckling [15].

At the endocytic actin patch, a small area of the flat plasma membrane invaginates towards the cytoplasm upon assembly of actin. In budding yeast, the invagination elongates up to 140nm in depth, and then is pinched off, releasing a tear-shaped vesicle [16]. Actin is essential for many of these steps, from the initiation of invagination to vesicle scission [17–19]. Despite extensive experimental work that characterized the overall dynamics of assembly, disassembly and ensemble movements of proteins of the actin meshwork [19–26], the precise structural organization of actin filaments within the endocytic patch remains unknown. Indeed, individual filaments are not resolvable even in electron micrographs, in which the actin network appears as a ribosome-exclusion zone, which is about 200nm in depth and 100nm in width [16].

Actin crosslinking proteins play a crucial role in determining the mechanical responses of the actin network to force perturbation [27–29]. Fimbrin (Fim1p) is the second most abundant protein recruited to the endocytic patch during clathrin-mediated endocytosis in fission yeast, after actin [20]. It has two actin binding domains that enable it to crosslink adjacent filaments. Deletion of fimbrin results in significant defects in endocytic internalization [24, 30, 31]. *In vitro* experiments have shown that fimbrin efficiently bundles long actin filaments, but bundling efficiency is reduced in the presence of capping protein as a result of decreased filament length [31]. It remains unclear how this length-dependent bundling activity arises and how this activity is related to the role of fimbrin during clathrin-mediated endocytosis.

Internalization of the endocytic membrane is hindered by the high turgor pressure (*P* ∼ 0.8 × 10^6^ Pa [32, 33]) inside yeast cells [34]. Under such high pressure, theoretical studies suggest that the force needed to initiate membrane invagination is on the order of 3000pN [35, 36] and actin polymerization is thought to provide the driving force. However, assuming no more than 150 filaments are simultaneously generating the force [11, 20, 21], each of these filaments has to generate a force of at least 20pN, an order of magnitude larger than the maximum polymerization force of ∼1pN of actin filaments measured *in vitro* [37]. This number of 20pN is likely an underestimate since the calculation here uses a very generous estimate for the number of growing filaments, up to 20-fold of what mathematical modeling predicts [11]. Therefore, actin polymerization alone is not enough to provide the force necessary to elongate a clathrin-coated pit. Even though type-I myosins participate in endocytosis, their low power output over a narrow range of forces suggest that they are more likely force-sensing tethers rather than force generators [38–40].

In this paper, we present a computational model for dynamic crosslinking of rigid actin filaments in conditions relevant to clathrin-mediated endocytosis. We show that kinetic and mechanical properties of the crosslinkers finely tune the structural transition of actin network between bundles and meshworks. In addition, we show that the chemical binding energy is converted into elastic energy upon binding of crosslinkers. The elastic energy stored in individual crosslinkers is significantly higher than their thermal energy. This surprising property is a consequence of the helical pitch of actin filaments, which leads to torsional strains between crosslinkers attached to a common pair of filaments. We discuss the mechanical implications of these torsionally stressed crosslinkers and propose a possible mechanism to generate directed rotation of filaments by orderly detaching the crosslinkers.

## Results

### Model of crosslinked rigid actin filaments

**Fig 1:**
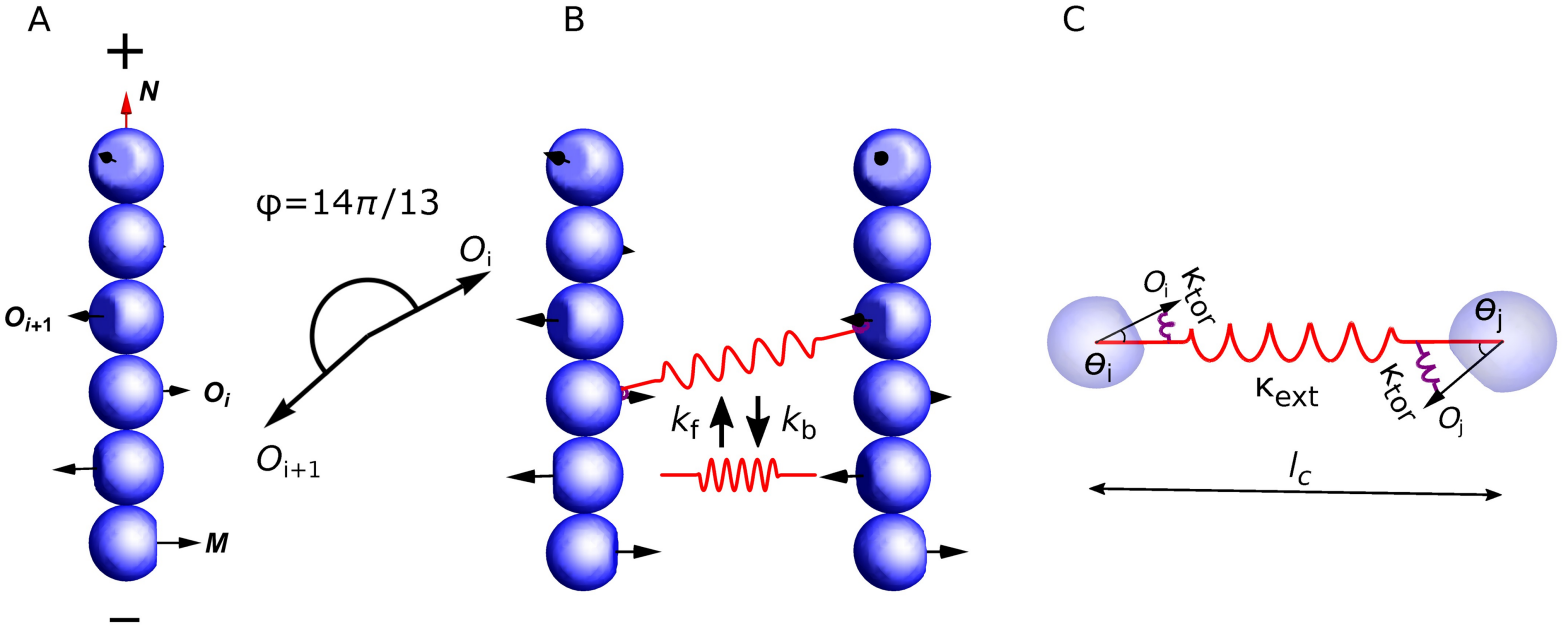
Model description. (A) Actin filaments are modeled as rigid rods made of subunits carrying an orientational vector **O**_*i*_ that represents the normal vector to the binding surface of the actin crosslinker. For visualization, each subunit is depicted as a sphere but is actually a disk-shape with a diameter of *b* = 6nm and a height of *δ* = 2.7nm. The orientation of a filament is described by the unit vector **N**, pointing from the pointed end (-) to the barbed end (+), and the first subunit’s orientational vector **M** = **O**_1_. Two consecutive subunits have an angle of *π*14/13 in their orientations. (B) Crosslinker turnover is described by stochastic formation and breakage of bonds between two actin subunits in different filaments, with rate constants *k*_f_ and *k*_b_, respectively. (C) Each crosslinker is modeled as a combination of three springs, one extensional spring with stiffness *κ*_ext_, which characterizes the stretchiness *l*_*c*_ between the two actin binding domains, and two torsional springs with stiffness *κ*_tor_, which characterizes the flexibility of the angles *θ*_*i*_ and *θ*_*j*_ between the axis of the crosslinker (which links the centers of each subunits it is attached to) and the vector normal to the binding surface of each actin subunit in each filament (**O**_*i*_ and **O**_*j*_).

We model actin filaments as rigid cylindrical rods with subunits that carry a helical pitch (Fig 1A). The length of filaments is restricted to the range of actin filament size during endocytosis (81nm to 216nm), around 2 orders of magnitude shorter than their persistence length ∼10*μ*m. Filaments of this length scale remain virtually straight under ∼10pN of force and untwisted under ∼100pN ⋅ nm of torque (see the Methods section). Therefore we can neglect bending and twisting, and describe the motion of a filament by its translational velocity **V***_c_* of center of mass, and angular velocity **Ω** relative to the center of mass. Details of the model can be found in the **Methods** section.

We model actin crosslinkers as elastic springs that connect two actin subunits in different filaments. Each spring has three elastic components: one in extension, which represents how much the spring is stretched, and two in torsion, each one representing how much the orientation of the axis of the crosslinker linking both actin subunits deviates from the vector normal to the binding interface of each actin subunit (Fig 1C). This elasticity is the simplest model to account for: (i) the intra-molecular flexibility between both actin binding domains, and (ii) the flexibility in the binding interface between each actin binding domains and the actin subunits they are bound to. Specifically, the elastic energy *E* of a crosslinker is composed of an extensional part *E*_ext_ and a torsional part *E*_tor_, *E* = *E*_ext_ + *E*_tor_. The extensional energy accounts for (i),

$$\begin{array}{c} E_{\text{ext}}=\frac{1}{2}\kappa_{\text{ext}}{\left(l_{c}-l_{0}\right)}^{2}, \end{array}$$ (1)

where *κ*_ext_ denotes the extensional stiffness, *l*_*c*_ denotes the length, and *l*_0_ denotes the rest length of the crosslinker (Fig 1C). The torsional energy accounts for (ii),

$$\begin{array}{c} E_{\text{tor}}=\frac{1}{2}\kappa_{\text{tor}}\left(\theta_{i}^{2}+\theta_{j}^{2}\right), \end{array}$$ (2)

where *κ*_tor_ denotes the torsional stiffness, *θ*_*i*_ and *θ*_*j*_ denote the angles between the actin subunits and the axis of the crosslinker (Fig 1C). Note that our model does not take into account the stiffness of the rotation of filaments around the axis of the crosslinker. Doing so would force us to consider a preferred relative orientation of the filaments with each other, and strongly favor either bundles or meshworks. No experimental values for the extensional and torsional stiffnesses of fimbrin are available in the literature. For our simulations, we used values within a few orders of magnitude of stiffnesses measured for fascin and antibodies [41, 42]. Crosslinker turnover is modeled as Poisson processes with a crosslinker formation rate constant *k*_f_ and a breakage rate constant *k*_b_, which increases exponentially with the total elastic energy (Fig 1B). A more detailed description is presented in the **Methods** section.

The length of filaments is kept constant in a given simulation and the total number of actin subunits in the filaments is fixed to *N*_actin_ = 7000 for all simulations [20]. The maximum occupancy of crosslinkers on filaments is constrained to remain under 25% (or 1 crosslinker for 4 subunits), which is equivalent to a maximum of 875 attached crosslinkers, close to the peak value ∼900 measured experimentally [20]. We initiate each simulation with uncrosslinked filaments that are randomly positioned and oriented. Reflecting boundary conditions are imposed to ensure filaments stay in a cubic box of 500nm in size. Simulations are performed using the reference values listed in Table 1 unless otherwise mentioned.

**Table 1:** List of parameters.

Symbol	Meaning	Reference value	Varied Range	Unit	Reference
*L*	Filament length	135	81-216	nm	[11]
*b*	Filament diameter	6	6	nm	[43]
*δ*	Length elongation of filaments by incorporation of a subunit	2.7	2.7	nm	[43]
*κ*_ext_	Extensional stiffness of the crosslinker	0.1	0.01-10	pN/nm	[41]
*κ*_tor_	Torsional stiffness of the crosslinker	10	0.1-100	pN ⋅ nm ⋅ rad^−1^	[42]^a^
*l*_0_	Rest length of the crosslinker	10	10	nm	[44]
*k*_f_	Rate constant of crosslink formation	1	0.01-1	s^−1^	[31, 45–47]
$k_{b}^{0}$	Strain-free rate constant of crosslink breakage	10	0.01-100	s^−1^	[31, 45–47]^b^
*E*_c_	Critical energy of the crosslinker	10	10	*k*_B_*T*	[48]
*r*_c_	Reaction distance between actin subunits for crosslinker binding	20	20	nm	*
*f*_st_	Magnitude of the repulsive force between filaments	100	100	pN	**
*k*_B_*T*	Thermal energy unit at room temperature	4.1	4.1	pN ⋅ nm	[49]
*N*_actin_	Total number of actin subunits	7000	7000		[20]
*η*	Viscosity of the medium	10	0.1-10	Pa ⋅ s	***
Δ*t*	Simulation time step	10^−5^	10^−7^−10^−5^	s	

*^a^* The torsional stiffness of a protein-antibody complex measured in Ref. [42] is ∼10^3^ pN/nm. We believe this value is an upper-bound for filament-crosslinker torsional stiffness.

*^b^* The strain-free detachment rate of fimbrin measured in Ref. [31] is ∼0.04 s^−1^. We studied this regime in (S3 Fig) but the reference value was chosen to demonstrate the phase transitions at higher detachment rates.

* We chose a value that is 2-fold the rest length.

** This force was tested to be large enough to avoid filaments crossing each other.

*** The reference value is on the higher end, and was chosen to minimize computational times. Our results did not change significantly for smaller viscosity (S7 Fig).

To quantify the organisation of actin filaments, we introduced the global and local nematic order parameters *S*_global_ and *S*_local_. These quantities characterize the degree of alignment between filaments in the entire simulation space, or in a local neighborhood, respectively. Their values range from 0 to 1, and a larger value indicates that filaments are more aligned with each other globally, for *S*_global_, or locally, for *S*_local_. Note that, in practice, the minimum reachable value for *S*_local_ is usually close to 0.4 (S6 Fig). The detailed mathematical definition of these parameters can be found in the **Methods** section.

Actin assembly and disassembly takes tens of seconds during endocytosis. We set the total simulation time to be 50s. Within this period, in most of our simulations, the metrics reach steady state. However, there are cases where the system is in a transient state, mostly due to slow convergence of the global nematic order parameter *S*_global_ (S8 Fig). Therefore, we chose the local nematic order parameter *S*_local_ averaged from 40s to 50s as the major metric to characterize the organisation of actin networks. Conclusions based on *S*_local_ are more robust than based on *S*_global_.

### Long filaments form bundles and short filaments form meshworks

**Fig 2:**
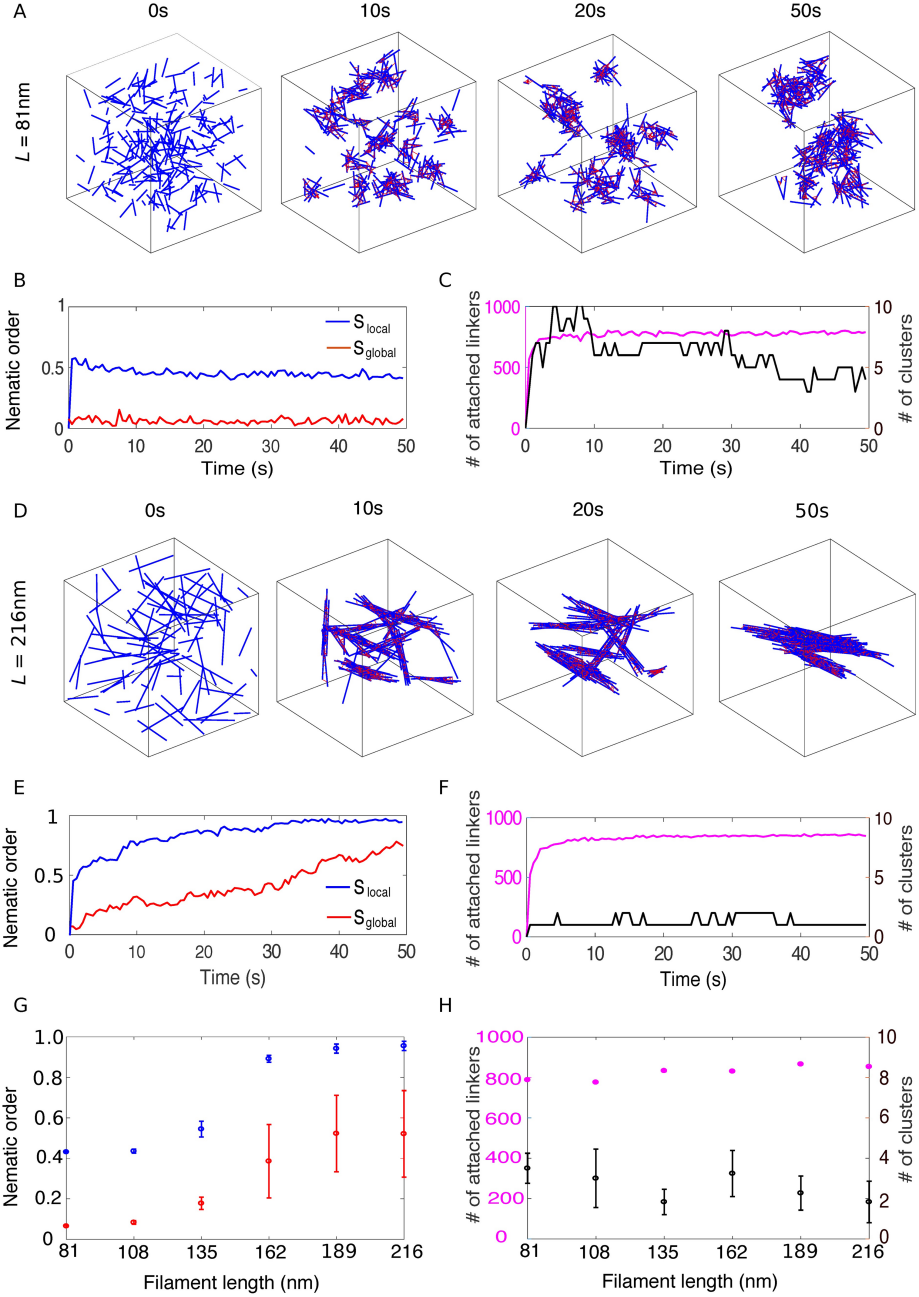
The structure of crosslinked actin networks depends on filament length. (A) Snapshots of an actin network formed by 81nm-long filaments in a 500nm-wide cubic box. Each filament is represented by a blue line and each crosslink by a red line. (B) Local (blue) and global (red) nematic order parameters of the actin network over the course of the simulation shown in (A). (C) Number of attached crosslinkers (magenta, left axis) and number of clusters (black, right axis) for the simulation in (A). (D-F) Similar figures as in (A-C) but for 216nm-long filaments. (G) Local (blue) and global (red) nematic order parameters as a function of filament length. (H) Number of attached crosslinkers (magenta, left axis) and number of clusters (black, right axis) as a function of filament length. In (G) and (H), for each simulation, the means of the metrics were calculated from the data between 40s to 50s and the error bars indicate standard deviation over 10 simulations.

We first studied how filament length influences the structure of actin clusters. For crosslinking rates *k*_f_ below 0.1*s*^−1^, the number of attached crosslinkers remained small, and actin filaments did not organize into higher order assemblies (S1 Fig). When the crosslinking rate *k*_f_ was high enough, initially disconnected short filaments (81nm) quickly formed small clusters that eventually coalesced into three to four larger clusters (Fig 2A). The number of attached crosslinkers rapidly saturated to a dynamic steady-state (Fig 2C), as the crosslinkers underwent constant turnover. Filaments within the cluster are organized into a disordered meshwork, which is characterized by a small local nematic order parameter (*S*_local_ ∼ 0.4, Fig 2B). Long filaments (216nm) rapidly aligned with their neighbors into small bundles (Fig 2D), as indicated by the fast convergence of the local nematic order parameter *S*_local_ to its steady state value around 1 (Fig 2E). Throughout the simulation, these locally aligned filaments remained connected with each other, and the number of clusters remained small (Fig 2F). These connected bundles slowly adjusted their orientations to eventually coalesce into a few large bundles.

When filament length was increased from 135nm to 162nm, the structure of the actin network transitioned from meshwork to bundle, indicated by the sharp increase of the local nematic order parameter *S*_local_ from ∼0.5 to above 0.9 (Fig 2G). The global nematic order parameter *S*_global_ showed similar trend as *S*_local_, but with a smaller magnitude, since filaments formed 2 to 4 independent clusters (Fig 2H). Altogether these results show that crosslinked actin filaments with a size and crosslinker density comparable to what is measured during endocytosis can self-organize into either meshworks (for short filaments) or bundles (for long filaments), and the phase transition is tightly controlled by filament length.

### Crosslinkers with high extensional stiffness drive bundle formation

**Fig 3:**
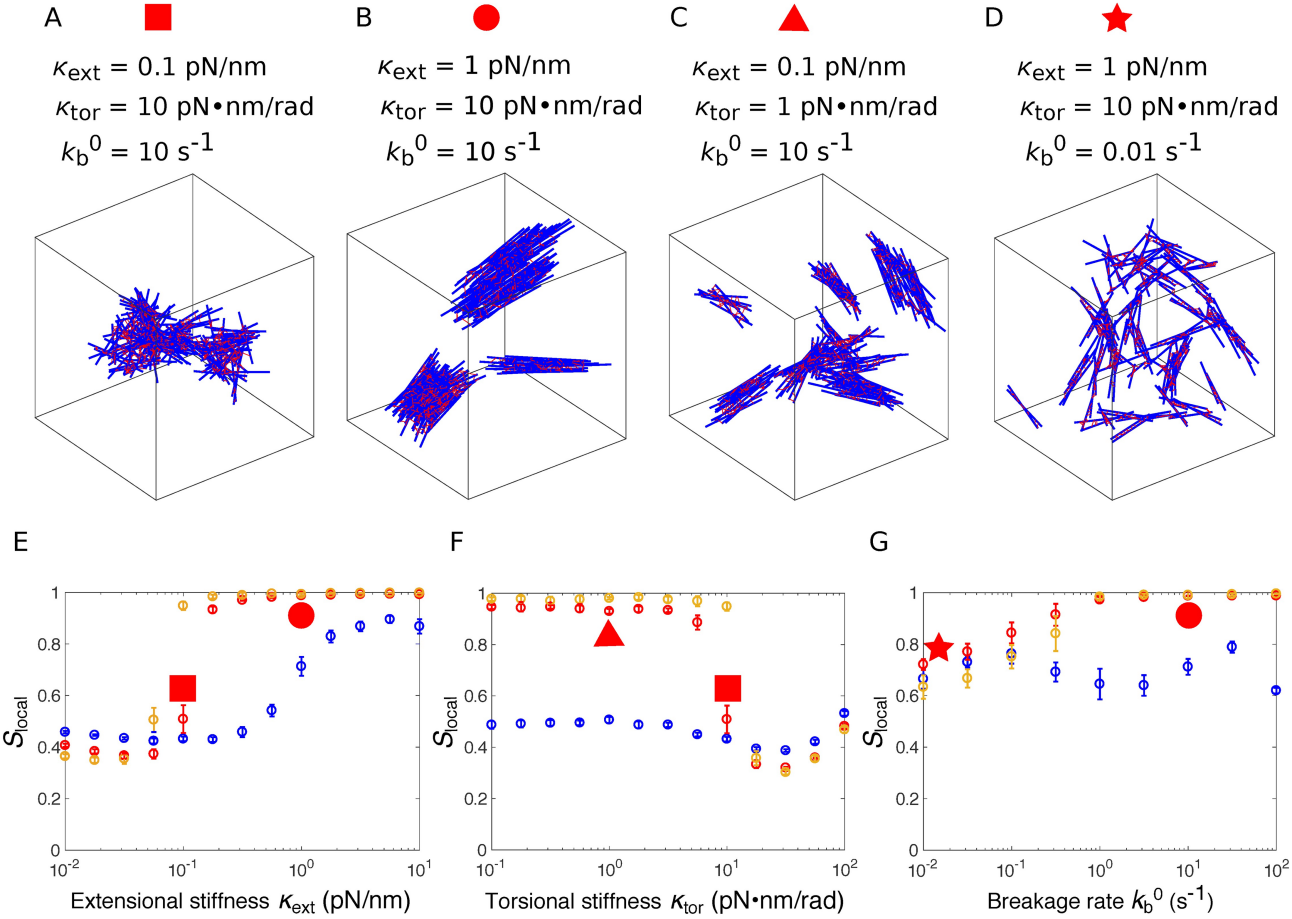
Influence of the crosslinker’s mechanical and kinetic properties on the organization of the actin network. (A-D) Organization of 135nm-long actin filaments at the end of the simulation (*t* = 50*s*) for different values of extensional stiffness *κ*_ext_, torsional stiffness *κ*_tor_, and breakage rate $k_{b}^{0}$, as indicated above the figure. (E) Local nematic order parameter *S*_local_ as a function of the extensional stiffness *κ*_ext_, with *κ*_tor_ = 10pN ⋅ nm ⋅ rad^−1^ and $k_{b}^{0}=10s^{-1}$. (F) Local nematic order parameter *S*_local_ as a function of the torsional stiffness *κ*_tor_, with *κ*_ext_ = 0.1pN/nm and $k_{b}^{0}=10s^{-1}$. (G) Local nematic order parameter *S*_local_ as a function of the strain-free breakage rate $k_{b}^{0}$, with *κ*_tor_ = 10pN ⋅ nm and *κ*_ext_ = 1pN/nm. In (E-G), simulations were performed for filaments of various lengths: 81nm (blue), 135nm (red), 189nm (orange). For each simulation, the means of *S*_local_ were calculated from the data between *t* = 40*s* to 50*s* and the error bars indicate standard deviation over 10 simulations. *S*_local_ corresponding to the networks in panels (A-D) are identified by the red symbols with corresponding shapes.

Next, we explored the influence of the mechanical properties of actin crosslinking proteins on the organization of actin filaments. The organization of medium length filaments (135nm) varied dramatically for different combinations of *κ*_ext_ and *κ*_tor_ (Fig 3A–3C). For low extensional stiffness *κ*_ext_, actin filaments organized into a meshwork (Fig 3A), while they formed bundles for higher *κ*_ext_ values (Fig 3B). The transition between meshwork and bundle was tightly controlled, since the local nematic order parameter had a sharp increase around *κ*_ext_ = 0.1pN/nm (Fig 3E, red) at given *κ*_tor_ = 10pN ⋅ nm ⋅ rad^−1^. This sharp transition was even more pronounced for longer filaments (189nm), as reported in both local and global nematic order parameters (Fig 3E and S2A Fig, orange). In contrast, when filaments were short (81nm), the transition was relatively smooth (Fig 3E, blue).

From the above results, we conclude that crosslinkers with large extensional stiffness favor bundle formation. This result can be intuitively explained by the following simplified but heuristic example involving only two filaments. If two filaments are initially aligned with each other, a slight change in orientation between both filaments results in the stretching of crosslinkers bound at different positions, which leads to a restoring torque to realign the filaments. The torque is proportional to the extensional stiffness and the distance between the positions of attached crosslinkers, thus stiffer crosslinkers create larger realignment torque than softer crosslinkers. Longer filaments not only have more crosslinkers, but also crosslinkers that are more distantly positioned, therefore more extended and more inclined to create a larger torque that will restore the parallel alignment of filaments.

### Crosslinkers with high torsional stiffness disfavor bundle formation

We next investigated the impact of torsional stiffness *κ*_tor_ on the organization of actin networks, keeping the extensional stiffness at a relatively small value (*κ*_ext_ = 0.1pN/nm). Torsional stiffness had virtually no influence on the organization of short filaments (81nm) (Fig 3F, blue). However, medium length filaments (135nm) had a sharp transition from bundle to meshwork at *κ*_tor_ = 10pN ⋅ nm ⋅ rad^−1^ (Fig 3F, red). A similar trend was also observed for long filaments (189nm) (Fig 3F, orange). At highest torsional stiffness tested (*κ*_tor_ = 100pN ⋅ nm ⋅ rad^−1^), crosslinker attachment lifetime was extremely short because their torsional energy often became much larger than the critical energy *E*_*c*_ that modulates the detachment rate (see Eq 16), therefore the number of attached linkers was significantly reduced (S2H Fig). The uncrosslinked actin network formed at this regime was different from the connected meshwork formed at *κ*_tor_ = 10pN ⋅ nm ⋅ rad^−1^, though their nematic order parameters *S*_local_ were similarly low.

The above results show that crosslinkers with high torsional stiffness disfavor bundle formation. This result can be explained by the fact that, when torsional stiffness is high, formation of several crosslinks between two aligned filaments results in very high torsional energies, due to the frustrated interactions between crosslinkers. This will become clear later in the paper. Therefore, it is energetically more favorable to form a few but over-stretched crosslinkers with many distant filaments than to form many but under-stretched crosslinkers with a few proximal filaments. In the former situation, filaments form a highly entangled actin meshwork. This explanation is supported by the decreasing number of clusters (S2E Fig), as well as the increasing extensional strains with *κ*_tor_ in the neighbourhood of *κ*_tor_ = 10pN ⋅ nm ⋅ rad^−1^ (S4C Fig).

### Turnover of crosslinkers is necessary for large bundle formation

We have shown that high extensional stiffness of crosslinkers favors bundle formation. When the breakage rate $k_{b}^{0}$ was reduced to 0.01*s*^−1^, even for large extensional stiffness (1pN/nm), filaments formed a structure where small bundles were interconnected but did not align with each other (Fig 3D), as indicated by the relatively low local nematic order parameter at $k_{b}^{0}=0.01s^{-1}$ compared with *S*_local_ at $k_{b}^{0}=10s^{-1}$ (Fig 3G). The difference in the global nematic order parameters *S*_global_ was even more pronounced for long filaments (189nm) (S2C Fig, orange). At very high breakage rate, the lifetime of bonds between filaments was so short that filaments formed an essentially random, uncrosslinked network. Altogether, we conclude that crosslinker turnover is essential for bundle formation, as alignment of bundles requires the breaking of the bonds that disfavor filament alignment.

### Phase diagram of filament organization

**Fig 4:**
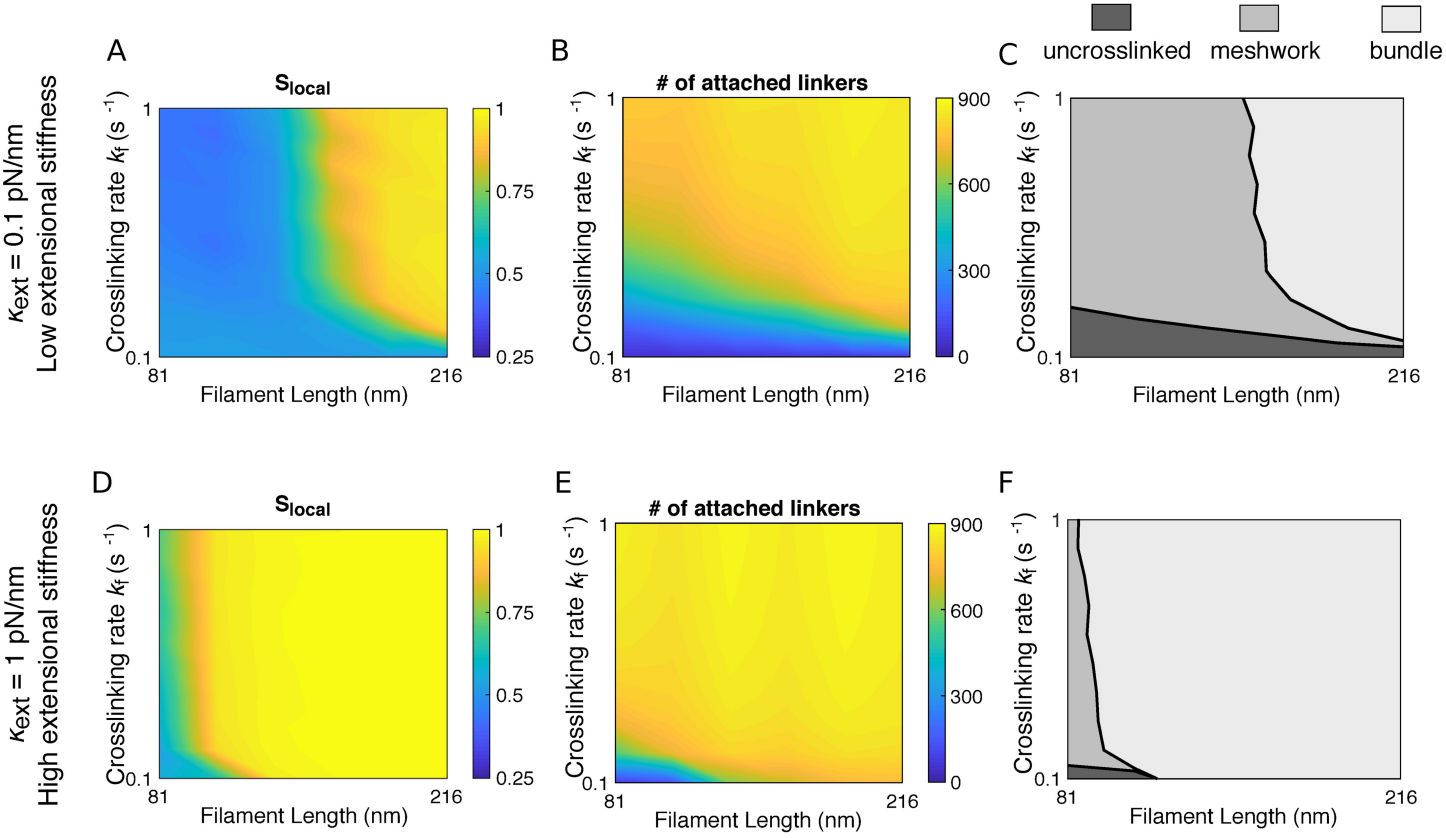
Phase diagram of actin network organization as a function of the crosslinking rate *k*_f_ and filament length *L*. (A, D) Local nematic order parameter *S*_local_ as a function of *k*_f_ and *L*. (B, E) Number of attached crosslinkers *N*_attach_ as a function of *k*_f_ and *L*. (C, F) Classification of actin network organizations as a function of *k*_f_ and *L*. The extensional stiffness *κ*_ext_ is 0.1pN/nm in panels (A-C), and 1pN/nm in panels (D-F). In panels (A, B, D, E), plots are constructed by interpolation of results obtained for increment Δ*L* = 27nm of *L* between 81nm and 218nm, and increment Δ*k*_f_ = 0.1*s*^−1^ of *k*_f_ between 0.1*s*^−1^ and 1*s*^−1^. The value for each parameter set is an average over 10 simulations. In (C) and (F), the border separating bundle (light gray) from meshwork (gray) is defined by *S*_local_ = 0.75. The border separating meshwork (gray) from uncrosslinked (dark gray) is defined by *N*_attach_ = 300.

Parameter dependence of the actin network structure is summarized in Fig 4 where we plotted the local nematic order parameter *S*_local_ and the number of attached crosslinkers *N*_attach_ as a function of crosslinking rate constant *k*_f_ and filament length *L*. For a combination of low extensional and high torsional stiffnesses (*κ*_ext_ = 0.1pN/nm, *κ*_tor_ = 10pN ⋅ nm ⋅ rad^−1^), values of *S*_local_ are concentrated either close to 1 or close to 0.5 (Fig 4A, yellow and blue regions, respectively). These two regions are separated by a narrow transition band around *S*_local_ = 0.75 (Fig 4A, green). Values of *N*_attach_ are clustered either close to the saturation number 875, or less than 100, divided by a transition band around *N*_attach_ = 300 (Fig 4B, yellow, blue and green regions, respectively). Therefore we chose the lines *S*_local_ = 0.75 and *N*_attach_ = 300 as the boundaries to define the phase diagram (Fig 4C). These two lines divide the parameter space into three regions: (1) Above the line *S*_local_ = 0.75, actin filaments are locally aligned into a bundle; (2) Between the lines *S*_local_ = 0.75 and *N*_attach_ = 300, filaments form a crosslinked, disordered meshwork; (3) Below the line *N*_attach_ = 300, filaments are essentially uncrosslinked over the entire simulation time. For larger extensional stiffness (*κ*_ext_ = 1pN/nm), the relative positions of the regions are similar, but most of the phase diagram corresponds to bundles (Fig 4F), and only the shortest filaments (81nm) form meshworks. In all cases, the existence of three regions in these phase diagrams requires moderate to high breakage rates. When the breakage rate is low ($k_{b}^{0}=0.01s^{-1}$), meshworks occupy the entire parameter space (S3 Fig).

### Crosslinked actin networks store elastic energy

**Fig 5:**
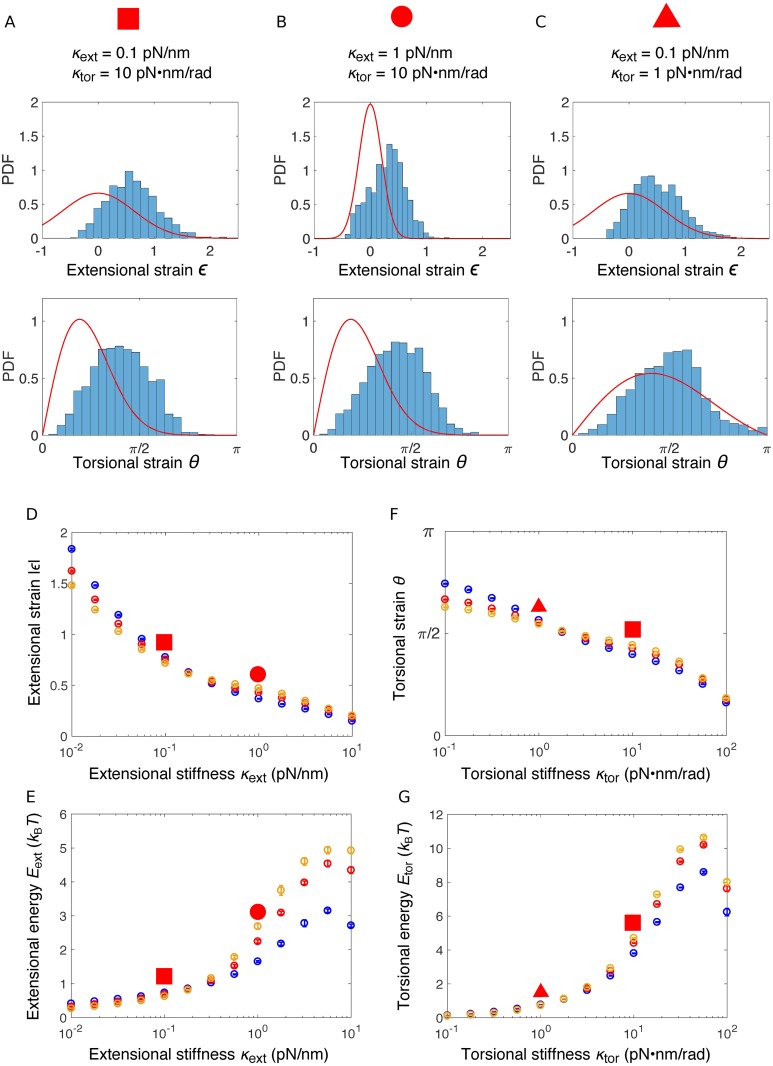
Straining of crosslinkers and energy storage. (A-C) Probability density distribution (PDF) of the extensional strains (top) and torsional strains (bottom) of crosslinkers with various stiffness values as indicated above the figure, at the end of individual simulations (*t* = 50*s*). For comparison, red lines indicate the corresponding Boltzmann distributions of a free spring with the same stiffness, $P\left(\epsilon \right)=C_{1}e^{-\frac{\kappa_{\text{ext}}l_{0}^{2}\epsilon^{2}}{2k_{B}T}}$ and $P\left(\theta \right)=C_{2}\sin\theta e^{-\frac{\kappa_{\text{tor}}\theta^{2}}{2k_{B}T}}$, where *C*_1_ and *C*_2_ are the normalization constants for *ϵ* ∈ (−1, ∞) and *θ* ∈ (0, *π*) respectively. Note that for the extensional strain, when we calculate the Boltzmann distribution, the energy contribution from steric interactions, which lead to the empty region at highly negative strains in the histogram, is neglected. (D, E) Average absolute value of the extensional strain *ϵ* (D) and the corresponding extensional energy *E*_ext_ (E) as a function of the extensional stiffness *κ*_ext_. (F, G) Average torsional strain *θ* (F) and the corresponding torsional energy *E*_tor_ (G) as a function of the torsional stiffness *κ*_tor_. In (D-G), simulations are performed for filaments of various lengths: 81nm (blue), 135nm (red), and 189nm (orange). For each simulation, the means of the energy were calculated from the data between 40s to 50s and the error bars indicate standard deviation over 10 simulations. Energies corresponding to the networks in panels (A-C) are identified by the red symbols with corresponding shapes.

Though crosslinkers in our model were not active elements, we found that crosslinkers rapidly became stretched in length, and twisted in angle. To quantify the crosslinkers’ deformations, we introduced the extensional strain *ϵ* = (*l*_*c*_ − *l*_0_)/*l*_0_, which measures the relative change of a crosslinker’s length *l*_*c*_ from its rest length *l*_0_, and the torsional strain *θ*, which measures the angle between the crosslinker and its bound actin subunits. For all stiffness values, the distributions of the extensional strain *ϵ* and the torsional strain *θ* significantly deviated from the corresponding Boltzmann distribution for a single independent free spring (Fig 5A–5C). Strikingly, the extensional strain *ϵ* peaked at ∼0.5 but not zero (Fig 5A–5C, top), indicating that the crosslinkers were stretched on average. The distribution had a narrower width for higher extensional stiffnesses *κ*_ext_. The peak of torsional strain *θ* decreased with increasing torsional stiffness, while the widths of the distributions were essentially the same as in the Boltzmann distribution (Fig 5A–5C, bottom).

The deviation from the Boltzmann distribution can be primarily accounted by the coupling between crosslinkers that are attached to the same pair of filaments. Indeed, a simpler 1D example of the Brownian motion of two particles each subject to a spring follows similar properties (S5 Fig). In this example, each spring generates a force of −*κx* if the particle is displaced from the equilibrium position 0 to *x*. If the movement of the two particles were independent (S5A Fig), the joint distribution of their positions *x*_1_ and *x*_2_ would simply be the product of identical individual distribution *p*(*x*_1_, *x*_2_) = *g*(*x*_1_)*g*(*x*_2_), where $g\left(x\right)=\sqrt{\frac{\kappa }{2\pi k_{B}T}}e^{-\kappa x^{2}/2k_{B}T}$ denotes the Boltzmann distribution of an individual particle. However, if the movements of the two particles are coupled, for instance, subject to the constraint *x*_1_ − *x*_2_ = 2*x*_0_ (S5B Fig), the position distribution of particle 1 becomes *p*(*x*_1_) = *Cg*(*x*_1_)*g*(*x*_1_ − 2*x*_0_), where *C* is the normalization constant. Therefore particle 1 is displaced by *x*_0_ due to the coupling with particle 2. Similarly, if two rigid filaments are bound by several crosslinkers, the extensional and torsional strains of these crosslinkers are coupled, and this coupling gives rise to significant strains in the crosslinkers.

We then determined the dependence of the elastic energy stored in the crosslinkers on the crosslinker stiffness. At fixed torsional stiffness *κ*_tor_ = 10pN ⋅ nm ⋅ rad^−1^, the average magnitude of the extensional strain |*ϵ*| decreased with increasing extensional stiffness *κ*_ext_ (Fig 5D). However, the average extensional energy per crosslinker $E_{\text{ext}}=\frac{1}{2}\kappa_{\text{ext}}l_{0}^{2}\epsilon^{2}$ increased (Fig 5E), from ∼0.3*k*_B_*T* for soft extensional springs up to ∼5*k*_B_*T* for stiff ones, with only a weak dependence on filament length (Fig 5E). The average torsional energy per crosslinker reads $E_{\text{tor}}=\frac{1}{2}\kappa_{\text{tor}}\left(\theta_{i}^{2}+\theta_{j}^{2}\right)$, with *θ*_*i*_ and *θ*_*j*_ being the average torsional strains at the two actin subunits. They have the same magnitude *θ*. At fixed extensional stiffness *κ*_ext_ = 0.1pN/nm, *E*_tor_ increased with torsional stiffness *κ*_tor_ from ∼0.2*k*_B_*T* to up to ∼10*k*_B_*T*, with again a weak dependence on filament length (Fig 5G). Both extensional and torsional energies vary relatively smoothly with the corresponding stiffnesses over three orders of magnitude, which is in stark contrast with the sharp structural transition between meshwork and bundle when stiffnesses are varied over the same range (Fig 3E and 3F).

Compared with the increase of the extensional energy *E*_ext_ with *κ*_ext_ (∼8 − 17 fold), the increase of torsional energy *E*_tor_ with *κ*_tor_ (∼40 − 50 fold) was more pronounced. This was likely due to stronger coupling between torsional strains of crosslinkers than between extensional strains. Elastic energy plateaued and then slightly decreased for very high stiffnesses (*κ*_ext_ = 10pN/nm or *κ*_tor_ = 100pN ⋅ nm ⋅ rad^−1^), which was the consequence of higher detachment rate leading to a smaller number of attached linkers, thus reducing the frustrated interactions (S2 Fig).

### Possible mechanism for torque production by release of the stored elastic energy

**Fig 6:**
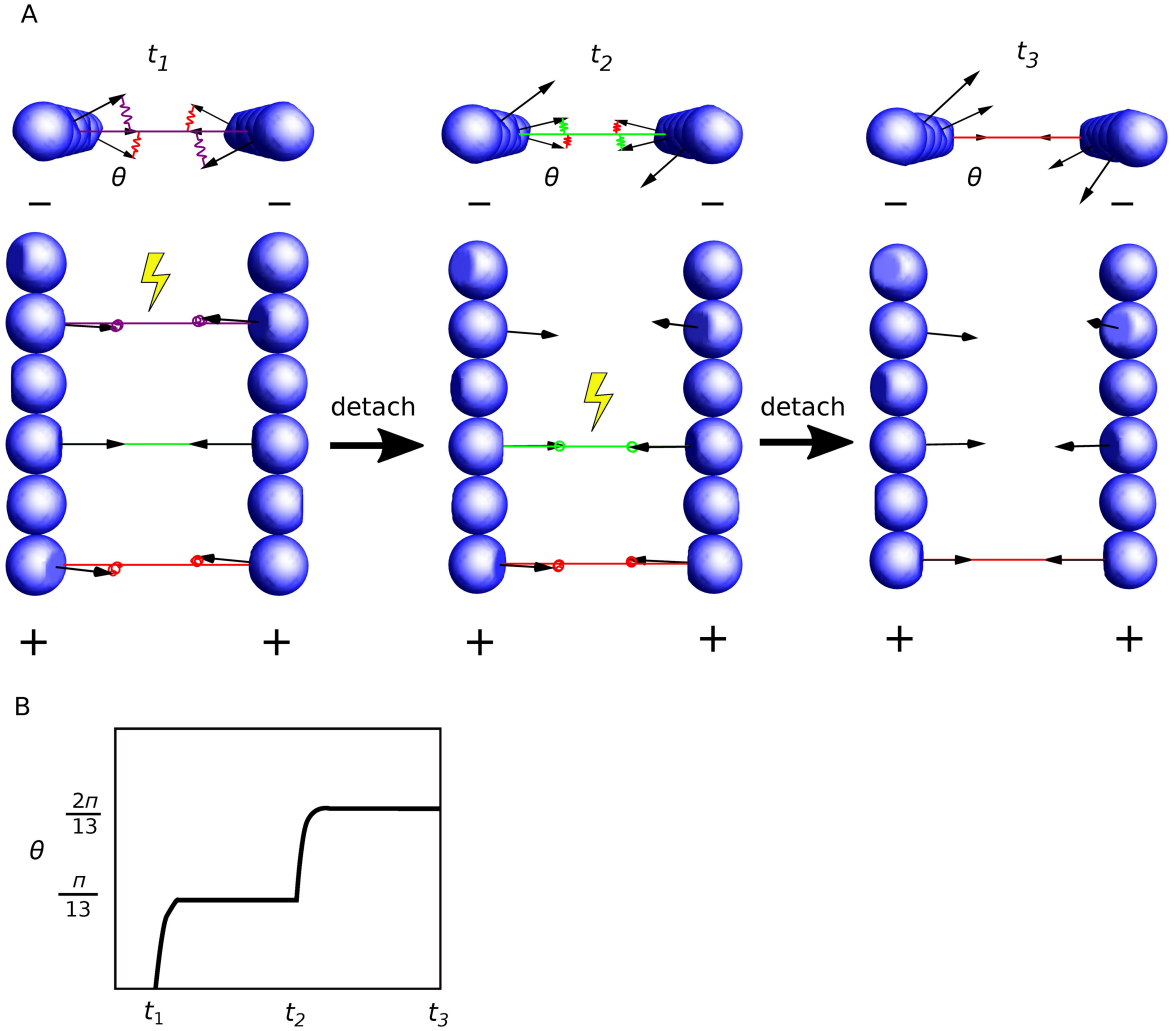
Schematic illustration of a possible mechanism for torque generation by sequential detachment of crosslinkers. (A) Two filaments in parallel are crosslinked on every other subunit. From left to right, crosslinkers are detached from the pointed end (−) to the barbed end (+) sequentially. Upon detachment of a crosslinker (yellow symbols), both filaments rotate around their axes counterclockwise. (B) The angular displacement of the filament upon every detachment is *π*/13.

We have shown that crosslinking of filaments leads to elastic energy stored in the crosslinkers. How can this energy be transformed into mechanical work? Here, we propose a mechanism for torque generation through orchestrated detachment of crosslinkers by studying a simple model with only two filaments. Let us consider a pair of short filaments, where every other subunit of each filament is crosslinked (Fig 6). For simplicity, we assume the two filaments are parallel and consider only the rotation of filaments around their axes. We show that consecutive detachment of crosslinkers from the pointed end to the barbed end lets the filament rotate in the same direction by *π*/13 for each detachment (Methods).

Building on this simple proof of principle, we show that sustained directional rotation can be achieved with any filament length and crosslinker spacing and configurations such that (i) the angles between two consecutive crosslinkers along the pair of filaments have the same sign, and (ii) the sum of all these angles is smaller than 2*π*. Under these conditions, breakage of crosslinkers from one end to the other produces directional torque (see Methods).

## Discussion

### Organization of actin filaments in diffraction limited assemblies

In this paper, we showed that highly crosslinked actin networks made of rigid filaments (< 200nm) can form either disordered meshworks or ordered bundles, depending on the filament length and the mechanical and kinetic properties of the crosslinkers. A recent *in vitro* study of short actin filaments (200nm) showed that with increasing density of filamin, the initially sparsely distributed actin filaments condensed into a spindle-shaped aggregate, in which the organization of actin filaments displayed nematic order [50]. This observation is consistent with the phase diagram in Fig 4C of our study for filaments of 200nm with increasing crosslinking rate.

We investigated the possible structures formed by actin filaments in the presence of elastic crosslinkers. However, our study does not take into account other actin regulating proteins involved in endocytosis, such as the Arp2/3 complex and capping protein, and thus does not completely resolve the question of the organization of filaments crosslinked with fimbrin at the site of endocytosis in yeast. Even with this limitation, these results provide valuable insights about possible actin filament architectures for endocytosis and other cellular processes that involve short actin filaments. Using the rate constants for fimbrin that have been measured *in vitro* [31, 45], and the fimbrin concentration in fission yeast cytoplasm (3.7 *μ*M) [20], these values correspond to rates in our model *k*_f_ = 0.2*s*^−1^ and $k_{b}^{0}=0.04s^{-1}$. Our simulations suggest that the slow off-rate of fimbrin should favor an assembly of actin filaments into a meshwork (S3 Fig). Further simulations with branched filaments, and with geometries and dynamics more representative of endocytosis will tell us which type of structure is present at endocytic sites. In addition, further experimental characterization of the mechanical properties of fimbrin and other crosslinkers will be key to understanding the self-organization of actin filaments in diffraction limited structures, and to test the predictions of our simulations.

### Mechanisms of energy storage by actin crosslinkers

Our simulations demonstrate that individual actin crosslinkers are able to store up to 10*k*_B_*T* of elastic energy, which is one order of magnitude higher than the elastic energy stored in an uncoupled spring in a thermal bath (1.5*k*_B_*T*), and about half of the energy released by ATP hydrolysis (∼25*k*_B_*T*).

To get a better sense of the amount of energy stored in the crosslinkers, one can make a comparison with the energy necessary to deform the plasma membrane into an endocytic vesicle. A back of the envelope calculation estimates the work needed to create a cylindrical invagination of *R*_*t*_ = 25nm in radius and *D*_*t*_ = 140nm in depth [16] against the turgor pressure *P* ∼ 0.8 × 10^6^ Pa is $P\pi R_{t}^{2}D_{t}=5.4\times 10^{4}k_{B}T$. The results of our model suggest that crosslinking filaments once with ∼900 crosslinkers, around 10^4^*k*_B_*T* energy could be stored, or about 1/6 of the total energy needed.

One may wonder where this large elastic energy comes from. We can show in a simplified model that the chemical binding energy of crosslinkers is indeed the source of the elastic energy. Let us consider two filaments with fixed positions and orientations, each having *N* subunits. In the following, we will consider the chemical balance between configurations where there is either *n* = 0 or 1 crosslinker between the two filaments. The rate at which a crosslinker is formed is *k*_f_Γ(1), where Γ(*n*) denotes the number of possible pairings to form *n* crosslinkers between subunits in the two filaments. The value of Γ(1) depends on the orientations and positions of the two filaments, and the mechanical properties of the crosslinkers, and could vary from 1 for very stiff crosslinkers and orthogonal filaments, to *N*^2^ for infinitely soft crosslinkers and parallel filaments. Here we assume Γ(1) = *N*, which implies that for each subunit in one filament, there is a unique subunit in the other filament that is within the reaction distance to allow a crosslinker to be formed. When one crosslink is formed, its detachment rate is $k_{b}^{0}e^{E/E_{c}}$, assuming the crosslinker bears an elastic energy *E*. If the attachment rate is greater than the detachment rate, the system is more likely to be crosslinked, even though the elastic energy *E* stored in the crosslinker tends to drive down the crosslinker occupancy. Noting $\mu_{\text{eff}}\equiv E_{c}\ln\left(Nk_{f}/k_{b}^{0}\right)$ the effective chemical binding energy that tends to drive up the occupancy of crosslinkers, this condition can be expressed as comparison between the effective chemical binding energy and the elastic energy *μ*_eff_ > *E*. Using the parameters $k_{b}^{0}=10s^{-1}$, *k*_f_ = 1*s*^−1^, *E*_*c*_ = 10*k*_B_*T* and *N* = 50, we estimate *μ*_eff_ = 16*k*_B_*T* for the first crosslink formation, which is larger than the 10*k*_B_*T* of elastic energy per crosslinker computed in our simulations. If we now consider the case where there are *n* crosslinkers formed between the filaments, the effective energy for binding an extra crosslinkers becomes $\mu_{\text{eff}}\left(n\right)=E_{c}\left[\ln\left(k_{f}/k_{b}^{0}\right)+\ln\left(\Gamma \left(n+1\right)/\Gamma \left(n\right)\right)\right]$. The second term in the bracket represents an entropic contribution that comes from the different ways of building *n* or *n* + 1 crosslinks between subunits of both filaments. This simplified two filament system illustrates how in our simulations with multiple filaments crosslinker occupancy is driven up by a similar entropic contribution in the chemical binding energy.

Our model considers “slip-bond” crosslinker detachements, i.e. crosslinkers are more likely to detach if force and torque are exerted on them (Eq 16). However, we could consider the case of “catch-bond” detachments, where crosslinkers are less likely to detach under force and torque, as it has been shown for some cytoskeleton proteins [40, 51, 52]. In this case, we expect that the elastic energy stored in crosslinkers would be larger than what we have observed in our simulations, and the conformational change required for the catch-bond behavior would increase the available energy limit.

The main reason energy storage is possible is that short actin filaments are rigid, which creates geometrical constraints on bound crosslinkers, forcing virtually all of them to fluctuate around average lengths and angles that are different from their rest lengths and angles. This implies that the crosslinkers are rigid enough to store elastic energy, but not as rigid as the filaments, so that filaments cannot be twisted or bent when crosslinked, or the distance over which filaments are twisted and bent in order to reduce the frustration, noted *L*_tb_, is much longer than the filament length *L*. In the opposite case, when filament length *L* ≫ *L*_tb_, filaments could form bundles in which individual filaments are twisted. Experiments conducted at this regime suggest that the frustrated interaction serves as a mechanism to control the size of the bundle [53] and cooperative binding of actin crosslinkers [54]. The two regimes have been theoretically studied by C. Heussinger and G. Grason [55].

### How can the stored energy be used productively in cellular processes?

Only a small fraction of the force necessary to deform the plasma membrane during clathrin-mediated endocytosis in yeast can be accounted for by actin polymerization alone. We predict that at least some of the missing force can come from the conversion of the elastic energy stored in the crosslinkers into force and/or torque.

In this paper, we proposed a specific mechanism for torque production by orchestrated detachment of crosslinkers. This mechanism is different from the Brownian ratchet mechanism of force production that is directly coupled to ATP hydrolysis [56, 57]. However, the ordered detachment has to be coupled to a non-equilibrium process to provide the information necessary for the ordered detachment. Treadmilling of filaments coupled to ATP hydrolysis could play such a role. To estimate the order of magnitude of free energy necessary to provide this information, let us consider a pair of short actin filaments (e.g. 50-subunit long) that are crosslinked by 10 crosslinkers. The free energy cost of detaching the crosslinkers in a specific order among all the 10! possibilities is ∼*k*_B_*T* ln 10! = 15*k*_B_*T*, which is only a small fraction of the energy provided by the ATP hydrolysis of two actin filaments undergoing treadmilling (50 × 25 = 1250*k*_B_*T*).

We stress that this ordered detachment is only one possible mechanism to use the energy and more mechanisms need to be discovered. Future work with more realistic models for endocytosis or other actin-based processes will likely uncover new orchestrated mechanisms for force production. In our system, crosslinkers are driven up to a mechanically pre-stressed state by chemical binding energy. In principle, in order to release the elastic energy stored in crosslinkers, change in energetics of crosslinker binding/unbinding is necessary to induce collective detachment of crosslinkers. When actin polymerization is considered, ATP-bound actin is incorporated at the barbed end and undergoes hydrolysis after incorporation. The nucleotide content change could alter the crosslinkers’ binding affinity, causing rapid detachment of crosslinkers, which is accelerated by the pre-stressed structure. Elastic energy released during this process could be converted into work by the reorganization of actin filaments.

### Conclusion

We developed a computational model to study the dynamic assembly of actin filaments mediated by elastic crosslinkers. The organization of actin filaments were classified into either a meshwork or a bundle, characterized by their nematic order parameter and the number of attached crosslinkers. We showed that the elastic energy stored in crosslinkers increased with their stiffness due to coupling between crosslinkers bound to rigid filaments. As a proof of principle, we showed that the elastic energy could be converted into mechanical work by orchestrated detachment of crosslinkers between two parallel filaments. Our work provides a new perspective to study the mechanisms of force and torque production by actin filaments, in addition to the traditional end polymerization. It also provides an alternative energy source to account for the insufficient force production by actin polymerization during clathrin-mediated endocytosis.

## Methods

### Model of actin filaments

Actin filaments are modeled as rigid cylindrical rods with diameter *b* and length *L*. The position of a filament is represented by its center of mass **C**. A unit vector **N** pointing from the filament’s pointed end to the barbed end indicates the orientation of the filament. The *i*-th subunit (counting from the pointed end) carries a unit vector **O**_*i*_, which is normal to the binding surface with a crosslinker (Fig 1A). We assume all **O**_*i*_-s are perpendicular to the filament’s orientation **N**. Based on the atomic structure of actin filaments [43], two consecutive subunits **O**_*i*_ and **O**_*i*+1_ span an angle of 14*π*/13 calculated counter-clockwise from **O**_*i*_ to **O**_*i*+1_. This means two consecutive subunits on different strands have their binding interface in almost opposite directions, and two consecutive subunits on the same strand have their binding interface at an angle of 2*π*/13 (28°). We arbitrarily choose the filament’s rotational vector **M** as the normal vector of the first subunit **M** = **O**_1_. Thus the orientational degree of freedom of the filament is fully captured by three orthonormal vectors **N**, **M** and **N** × **M**.

The motion of a filament is described by its translational velocity **V***_c_* and angular velocity **Ω**, which are defined by the following equations:

$$\begin{array}{ccc} \frac{dC}{dt} & = & V_{c}, \end{array}$$ (3)

$$\begin{array}{ccc} \frac{dN}{dt} & = & \Omega \times N, \end{array}$$ (4)

$$\begin{array}{ccc} \frac{dM}{dt} & = & \Omega \times M. \end{array}$$ (5)

The velocities **V***_c_* and **Ω** are governed by the force-balance and torque-balance equations:

$$\begin{array}{ccc} \Xi^{t}V_{c} & = & F^{e}+F^{s}, \end{array}$$ (6)

$$\begin{array}{ccc} \Xi^{r}\Omega & = & T^{e}+T^{s}. \end{array}$$ (7)

Here the 3 × 3 matrices *Ξ*^t^ and *Ξ*^r^ denote the frictional matrix associated with translational and rotational motion of the filament, respectively. The vectors **F**^e^ and **T**^e^ denote the total deterministic force and torque generated by crosslinkers or induced by steric interactions between filaments. The vectors **F**^s^ and **T**^s^ denote the stochastic force and torque, which obey the fluctuation-dissipation relations:

$$\begin{array}{ccc} \left\langle F_{i}^{s}\left(t\right)F_{j}^{s}\left(t^{\prime }\right)\right\rangle & = & 2k_{B}T\delta \left(t-t^{\prime }\right)\Xi_{ij}^{t}, \end{array}$$ (8)

$$\begin{array}{ccc} \left\langle T_{i}^{s}\left(t\right)T_{j}^{s}\left(t^{\prime }\right)\right\rangle & = & 2k_{B}T\delta \left(t-t^{\prime }\right)\Xi_{ij}^{r}. \end{array}$$ (9)

Here the subscript indicates the element of the vectors or matrices. The frictional matrices are anisotropic, and given by [58]:

$$\begin{array}{ccc} \Xi^{t} & = & \xi_{\|}^{t}N⊗N+\xi_{⊥}^{t}\left(I-N⊗N\right), \end{array}$$ (10)

$$\begin{array}{ccc} \Xi^{r} & = & \xi_{\|}^{r}N⊗N+\xi_{⊥}^{r}\left(I-N⊗N\right), \end{array}$$ (11)

where $\xi_{\|}^{t}$ and $\xi_{⊥}^{t}$ are the frictional coefficients for translational movement parallel with and perpendicular to the filament’s central axis, and $\xi_{\|}^{r}$ and $\xi_{⊥}^{r}$ are the corresponding frictional coefficients for rotation. The 3 × 3 identity matrix is denoted by **I**, and ⊗ denotes the outer product of two vectors. The anisotropic frictional coefficients depend on filament length *L* and diameter *b* via the relations [43]:

$$\begin{array}{c} \xi_{\|}^{t}=\frac{2\pi \eta L}{\ln(L/b)-0.2},\;\xi_{⊥}^{t}=\frac{4\pi \eta L}{\ln(L/b)+0.84}, \end{array}$$ (12)

$$\begin{array}{c} \xi_{\|}^{r}=\pi \eta b^{2}L,\;\xi_{⊥}^{r}=\frac{\pi \eta L^{3}}{3(\ln(L/b)-0.66)}. \end{array}$$ (13)

Here *η* denotes the viscosity of the medium.

To account for the steric interaction between filaments, if the shortest distance *r*_min_ between two filaments is less than the diameter *b* of a filament, a constant repulsive force *f*_st_ is applied along the lines connecting the two nearest points.

In each time step, we calculate all the forces and torques acting on a filament and determine the translational velocity **V**_c_ and angular velocity **Ω** of the filament according to Eqs (6) and (7). The center of mass of a filament is then updated as:

$$\begin{array}{c} C\left(t+\Delta t\right)=C\left(t\right)+V_{c}\Delta t. \end{array}$$ (14)

The updated orientations are:

$$\begin{array}{c} N(t+\Delta t)=\text{Rot}(\Omega \Delta t)N(t), \end{array}$$ (15)

where Rot (**Ω**Δ*t*) denotes the rotation matrix defined by the vector **Ω**Δ*t*. The rotation vector **M**(*t*) is updated in the same way.

### Model of actin crosslinking proteins

Each actin crosslinking protein is modeled as an elastic spring that bridges two actin subunits in two separate filaments. The crosslinking of two unoccupied subunits proceeds with a rate constant of *k*_f_, as long as the subunits are less than *r*_c_ apart. The breakage of an established crosslink is assumed to follow a “slip-bond” mechanism and occurs with an energy-dependent rate constant:

$$\begin{array}{c} k_{b}=k_{b}^{0}e^{E/E_{c}} \end{array}$$ (16)

where $k_{b}^{0}$ denotes the strain-free breakage rate constant, *E* denotes the total elastic energy, and *E*_*c*_ denotes the critical energy that determines the sensitivity of the bond breakage on the forces and torques.

The elastic energy *E* of a crosslinker that bridges actin subunits in filaments *α* and *β* is a function of the positions, orientations and rotations of both filaments, as well as its positions in the filament, *E* = *E*(**C**^*α*/*β*^, **N**^*α*/*β*^, **M**^*α*/*β*^). The force generated by the crosslinker on filament *α* reads:

$$\begin{array}{c} F^{\alpha }=-\frac{\partial E}{\partial C^{\alpha }}. \end{array}$$ (17)

To determine the torque generated by the crosslinker on filament *α*, we choose three orthnormal vectors **e**_1_, **e**_2_, **e**_3_ and virtually rotate filament *α* by an infinitesimal angle *ϕ*_*i*_ around the axis **e**_*i*_. These operations are equivalent to applying the following infinitesimal changes to the orientational vectors of filament:

$$\begin{array}{ccc} N^{\alpha } & \to & N^{\alpha }+\phi_{i}e_{i}\times N^{\alpha }, \end{array}$$ (18)

$$\begin{array}{ccc} M^{\alpha } & \to & M^{\alpha }+\phi_{i}e_{i}\times M^{\alpha }. \end{array}$$ (19)

The elastic energy correspondingly has an infinitesimal change *E* → *E* + Δ*E*. The torque then reads:

$$\begin{array}{c} T^{\alpha }=-\sum_{i=1}^{3}\frac{\partial \Delta E}{\partial \phi_{i}}e_{i} \end{array}$$ (20)

Forces and torques acting on filament *β* are derived in a similar way. The total elastic force and torque are obtained by summing (17) and (20) over all the crosslinkers bound to the filament.

### Events performed in a single simulation step

At each time step Δ*t* of the simulation, we perform the following operations:

1. Calculate all the deterministic and stochastic forces and torques on each filament. These forces and torques determine the translational and angular velocities of filaments according to Eqs (6) and (7). The positions and orientations of filaments are then updated according to Eqs (14) and (15).
2. For each pair of filament *α* and *β*, determine the number *N*_*a*_ of crosslinkers to be attached between them by drawing a random number from the Poisson distribution, $\text{Pois}(N_{\text{mon}}^{\alpha }N_{\text{mon}}^{\beta }k_{f}\Delta t)$, where $N_{\text{mon}}^{\alpha }$ and $N_{\text{mon}}^{\beta }$ are the number of actin subunits in each filament. Then randomly pick *N*_*a*_ pairs of subunits (*i*, *j*) with *i* in filament *α* and *j* in filament *β*. If they are not already occupied by a crosslinker and distance between them is shorter than the interacting distance *r*_*c*_ and the total number of already occupied subunits in each filament is less than $g_{\text{max}}N_{\text{mon}}^{\alpha /\beta }$, a crosslinker is built between the subunits (*i*, *j*).
3. For each already existing crosslinker, determine whether it will be detached by comparing a uniformly distributed random number *u* on the interval [0, 1] with the energy-dependent breakage rate *k*_b_ in Eq (16). If *u* < 1 − *e*^−*k*_b_Δ*t*^, the crosslinker is detached.

In our simulation, we always set the time step Δ*t* at least 100 times smaller than the relaxation time of the spring $\tau =\text{min}(\xi_{\|/⊥}^{t}/\kappa_{\text{ext}},\xi_{\|/⊥}^{r}/\kappa_{\text{tor}})$ to ensure that we correctly capture the dynamics of the springs. For computational reasons, we used a high viscosity value *η* = 10Pa ⋅ s, such that the relaxation time *τ* ∼ 0.01s for *κ*_ext_ = 0.1pN/nm and *κ*_tor_ = 10pN ⋅ nm ⋅ rad^−1^. We tested values of lower viscosity down to *η* = 0.1Pa ⋅ s. There is no significant difference in the local nematic order parameter *S*_local_ and the elastic energies *E*_ext_ and *E*_tor_ between *η* = 0.1Pa ⋅ s and *η* = 10Pa ⋅ s. However, the global nematic order parameter *S*_global_ for long filaments is increased to 1 at lower viscosity (S7 Fig). This is because the enhanced diffusion increases the probability of filaments moving close to each other. As a result, the separated bundles observed at high viscosity merge into a single bundle when the viscosity is low, increasing the global nematic order parameter.

### Metrics to describe local and global organization of filaments

We characterize the structure of actin clusters by introducing local and global nematic order parameter *S*_local_ and *S*_global_. We map the connections between filaments into an undirected graph, with filaments being the nodes, and the number of crosslinkers being the value of the edges connecting two nodes. Filaments in a connected component of the graph are said to form a cluster if the number of filaments in the component is more than 10. The nematic order parameter *S* for a group of filaments is the maximum eigenvalue of the following matrix [59]:

$$\begin{array}{c} Q=\frac{1}{G}\sum_{\alpha }\left(N^{\alpha }⊗N^{\alpha }-\frac{1}{3}I\right), \end{array}$$ (21)

where *G* denotes the number of filaments in the group, **N**^*α*^ denotes the orientational vector of filament *α*. For global nematic order parameter *S*_global_, the group in Eq (21) includes all the filaments. For a particular filament α*, $S_{\text{local}}^{\alpha^{*}}$ is defined by grouping the filament α* and its connected nodes in Eq (21). The local nematic order parameter *S*_local_ is the average of $S_{\text{local}}^{\alpha }$ over all the filaments that have at least 2 connected nodes.

Both *S*_local_ and *S*_global_ are in the range of [0, 1]. Values of *S*_local_ close to 1 indicate that filaments are locally aligned with their connected neighbors. Values of *S*_global_ close to 1 indicate that all the filaments are aligned. In general *S*_local_ is greater than *S*_global_, and reaches steady state more rapidly, because filaments that are in close proximity can rapidly align, but it takes time for distant clusters of filaments to collide and reorient. *S*_local_ is also more consistent over different simulations than *S*_global_, as reported by smaller error bars for *S*_local_ than for *S*_global_ (e.g. Fig 2G). If the viscosity of the medium *η* is reduced to 1Pa ⋅ s, filaments form a single cluster and the error bars of *S*_global_ become comparable with *S*_local_ (S7B and S7C Fig).

Note that in a sparsely connected network with filaments in random orientation, *S*_local_ ∼ 0.5 (S1B Fig) is higher than one should expect (∼0). This artifact is due to the fact that the sum in Eq (21) is done over a very small number of filaments (∼3). We confirmed this property by numerically calculating the nematic order parameter for three unit vectors with random orientations. The resulting distribution of *S*_local_ has a peak at 0.45 (S6 Fig). This almost uncrosslinked network should be distinguished from the densely connected actin meshworks, which have local nematic order parameters *S*_local_ in the same range (∼ 0.4 Fig 2B) but possess a large number of attached crosslinkers. Therefore, the number of crosslinkers in the meshwork is required to distinguish these two structures.

### Validity of the rigidity assumption

In our model, we assumed that filaments are rigid. This rigidity assumption implies that (i) thermal fluctuations, and (ii) forces and torques exerted by crosslinkers do not significantly bend or twist the filaments. We can verify a posteriori that these conditions are actually fullfiled in our simulations. Indeed, the maximum force produced by a crosslinker in our simulations is ∼10pN when the extensional stiffness *κ*_ext_ reaches 10pN/nm, and the maximum torque is ∼100pN ⋅nm when the torsional stiffness *κ*_tor_ reaches 100pN ⋅ nm ⋅ rad^−1^. Given the persistence length of actin filament for both bending and twisting is *L*_*p*_ ∼ 10*μ*m [12, 14, 60, 61], for a filament of length *L* = 200nm which consists of *N* = *L*/*δ* = 74 subunits, the angular change between two consecutive subunits due to thermal fluctuation is $\arccos\left(e^{-L/L_{p}}\right)/N=0.15^{{}^{\circ}}$. The angular change due to bending caused by a force of *f* = 10pN in the middle of the filament when the two ends are fixed is $\arctan\left(\frac{fL^{2}}{48L_{p}k_{B}T}\right)/N=0.15^{{}^{\circ}}$ [62]. The twisting angle by a torque of *T* = 100pN ⋅ nm is $\left(\frac{TL}{L_{p}k_{B}T}\right)/N=0.37^{{}^{\circ}}$. Therefore, it is safe to consider filaments as stiff, and the energy stored in crosslinkers would not be dramatically different even if the finite stiffness of filaments was taken into account.

### Direct rotation of filaments by consecutive detachment of crosslinkers

We consider the rotation of two parallel filaments around their axes by consecutive detachment of crosslinkers from the pointed end to the barbed end. We assume that every other subunit of each filament is crosslinked, such that the *i*-th crosslinker has an angle of

$$\begin{array}{c} \theta_{i}=\theta_{1}+\left(i-1\right)2\pi /13 \end{array}$$ (22)

with its attached actin subunit. The torque generated by the *i*-th crosslinker on the filament thus is −*κ*_tor_*θ*_*i*_. Here the crosslinker label *i* is ordered according to their distance to the pointed end of filaments. At torque balanced state, i.e., $\sum_{i=1}^{n}\theta_{i}=0$, we have *θ*_1_ = −(*n* − 1)*π*/13. Upon detachment of crosslinker 1, the total torque becomes imbalanced and the filament makes a rotation of angle Δ*ϕ* to reach a new torque balanced state, i.e., $\sum_{i=2}^{n}\left(\theta_{i}+\Delta \phi \right)=0$. This leads to Δ*ϕ* = −*π*/13. Similarly we can show that attachment of a new crosslinker at the (*n* + 1)-th position *θ*_*n*+1_ = *θ*_1_ + *n*2*π*/13 will cause the filament to rotate the same angle in the same direction as caused by detachment of the first crosslinker.

Note that even though the rotation angle Δ*ϕ* is independent of the number of attached crosslinkers, it is required that crosslinkers are present in large enough number or are stiff enough to ensure that rotation will be significantly larger than thermal fluctuations, i.e. $\frac{1}{2}\kappa_{\text{tor}}\theta_{1}^{2}\gg k_{B}T$. For instance, if the number of crosslinkers *n* = 10, this requires *κ*_tor_ ≫ 2pN ⋅ nm ⋅ rad^−1^.

The above calculation can be easily extended to situations with more relaxed conditions than Eq (22). The angular rotation Δ*ϕ*^(*i*)^ of the filament upon detachment of the *i*-th crosslinker satisfies the recursive relation:

$$\begin{array}{c} \Delta \phi^{\left(i\right)}=\frac{\theta_{i}+\sum_{j=1}^{i-1}\Delta \phi^{\left(j\right)}}{n-i}. \end{array}$$ (23)

Directed rotation requires that Δ*ϕ*^(*i*)^ have the same sign for all *i*. This condition is equivalent to considering that the angles between consecutive crosslinkers (*θ*_*i*_ − *θ*_*i*−1_) have the same sign for all *i*.

### Derivation of the effective binding energy *μ*_eff_

We consider a simplified model in which, when there are *n* crosslinkers formed, each crosslinker stores an elastic energy of *E*_*n*_. In fact, *E*_*n*_ is varied among different crosslinkers and dependent on their positions and orientations in the filaments. Here we assume *E*_*n*_ only depends on *n*, and the probability distribution *P*(*n*, *t*) for the number of crosslinkers is governed by

$$\begin{array}{ccc} \frac{dP(n,t)}{dt} & = & k_{f}\Phi \left(n|n-1\right)P\left(n-1\right)+\left(n+1\right)k_{b}^{0}e^{E_{n+1}/E_{c}}P\left(n+1\right)\\ & - & [k_{f}\Phi \left(n+1|n\right)+nk_{b}^{0}e^{E_{n}/E_{c}}]P\left(n\right), \end{array}$$ (24)

where Φ(*n*|*n* − 1) denotes the number of ways to build the *n*-th crosslinker, given there are already *n* − 1 crosslinkers formed. The steady state distribution reads

$$\begin{array}{c} P^{\text{ss}}\left(n\right)=P\left(0\right){\left(\frac{k_{f}}{k_{b}^{0}}\right)}^{n}\Gamma \left(n\right)e^{-\frac{1}{E_{c}}\sum_{i=1}^{n}E_{i}}, \end{array}$$ (25)

where $\Gamma \left(n\right)=\frac{1}{n!}\prod_{i=1}^{n}\Phi \left(i|i-1\right)$ is the number of possible ways to build *n* crosslinkers. By comparing the distribution of *P*^ss^(*n* + 1) with *P*^ss^(*n*), we have

$$\begin{array}{c} \frac{P^{\text{ss}}\left(n+1\right)}{P^{\text{ss}}\left(n\right)}=e^{-\frac{E_{n+1}-\mu_{\text{eff}}\left(n\right)}{E_{c}}}, \end{array}$$ (26)

where *μ*_eff_(*n*) is the effective binding energy defined in the text. If *μ*_eff_(*n*) > *E*_*n*+1_, the system is driven up to the (*n* + 1)-state, storing an elastic energy of *E*_*n*+1_.

## Supporting information

S1 Fig Filaments do not cluster if the crosslinker formation rate *k*_f_ is low. (A) Snapshots of an actin network with 81nm-long filaments in a 500nm-wide cubic box. The crosslinking rate is *k*_f_ = 0.1*s*^−1^. Each filament is represented by a blue line and each crosslink by a red line. (B) Evolution of the local (blue) and global (red) nematic order parameter of the actin network over the course of the simulation shown in (A). (C) Evolution of the number of attached crosslinkers (magenta, left axis) and the number of clusters (black, right axis) for the simulation in (A).
(TIF)

S2 FigInfluence of the crosslinker’s mechanical and kinetic properties on the organization of the actin network.(A-C) Global nematic order parameter as a function of the extensional stiffness *κ*_ext_ (A), the torsional stiffness *κ*_tor_ (B), and the linker breakage rate $k_{b}^{0}$ (C). (D-F) Number of clusters as a function of the same set of parameters. (G-I) Number of attached linkers as a function of the same set of parameters. Simulations are performed for filaments of various lengths: 81nm (blue), 135nm (red) and 189nm (orange). For each simulation, the means of the metrics were calculated from the data between 40s to 50s and the error bars indicate standard deviation over 10 simulations.(TIF)

S3 FigPhase diagram of the actin network organization with low strain-free breakage rate $k_{b}^{0}=0.01s^{-1}$ and high extensional stiffness *κ*_ext_ = 1pN/nm.(A) Local nematic order parameter *S*_local_ as a function of *k*_f_ and *L*. (B) Number of attached crosslinkers *N*_attach_ as a function of *k*_f_ and *L*. (C) Classification of actin network organizations as a function of *k*_f_ and *L*. Meshworks occupy the entire parameter space. The criteria delimiting each possible organization are the same as in Fig 4E and 4G, i.e. *S*_local_ = 0.75 and *N*_attach_ = 300.(TIF)

S4 Fig Straining of crosslinkers and energy storage. (A, B) Average torsional strain *θ* (A) and torsional energy (B) as a function of the extensional stiffness *κ*_ext_. (C, D) Average absolute value of the extensional strain |*ϵ*| (C) and extensional energy (D) as a function of the torsional stiffness *κ*_tor_. Simulations were performed for filaments of various lengths: 81nm (blue), 135nm (red), and 189nm (orange). For each simulation, the means of the metrics were calculated from the data between 40s to 50s and the error bars indicate standard deviation over 10 simulations.
(TIF)

S5 Fig Schematic illustration of the one-dimensional Brownian motion of two particles attached to each other with springs. The black dot on the spring is constrained to stay at position 0. (A) The movements of the two particles are independent. (B) The movements of the two particles are strongly coupled (by the black bar) and constrained such that *x*_1_ − *x*_2_ = 2*x*_0_. (C) The position distribution of the particle 1 is shown in the decoupled case (blue) and in the coupled case (orange).
(TIF)

S6 Fig Distribution of the nematic order parameter for three unit vectors with random orientations. This distribution shows that randomly oriented vectors have a nematic order parameter centered around 0.4, rather than 0. The distribution was drawn from 200 simulations.
(TIF)

S7 Fig Effect of viscosity on the structure of actin filaments and the elastic energy of crosslinkers. (A-C) Nematic order parameter as a function of filament length at various viscosities as indicated above the panel. Global and local nematic orders are shown in red and blue, respectively. (D-F) Elastic energy as a function of filament length at various viscosities as indicated above the panel. Extensional and torsional energies are shown in blue and red, respectively. The total simulation time is 10s for *η* = 0.1Pa ⋅ s, and is 50*s* for *η* = 1 − 10Pa ⋅ s. For each simulation, the means of the metrics were calculated from the data between 40s to 50s (for *η* = 1 − 10Pa ⋅ s) or between 8*s* and 10*s* (for *η* = 0.1Pa ⋅ s), and the error bars indicate standard deviation over 10 simulations.
(TIF)

S8 Fig Convergence test of the simulation. (A, C, E) Temporal evolution of local (blue) and global (red) nematic order parameter for filaments of length 81nm (A), 135nm (C) and 189nm (E). (B, D, F) Temporal evolution of number of attached crosslinkers for filaments of length 81nm (B), 135nm (D) and 189nm (F). All the metrics are averaged over 20 simulations for total 75s (A, B) or 150s (C-F).
(TIF)
