# Supplementary figures and images for: Structural organization and energy storage in crosslinked actin assemblies
Source: PLoS Comput Biol. 2018 May 29;14(5):e1006150. doi: 10.1371/journal.pcbi.1006150 (PMC5993335; doi:10.1371/journal.pcbi.1006150)

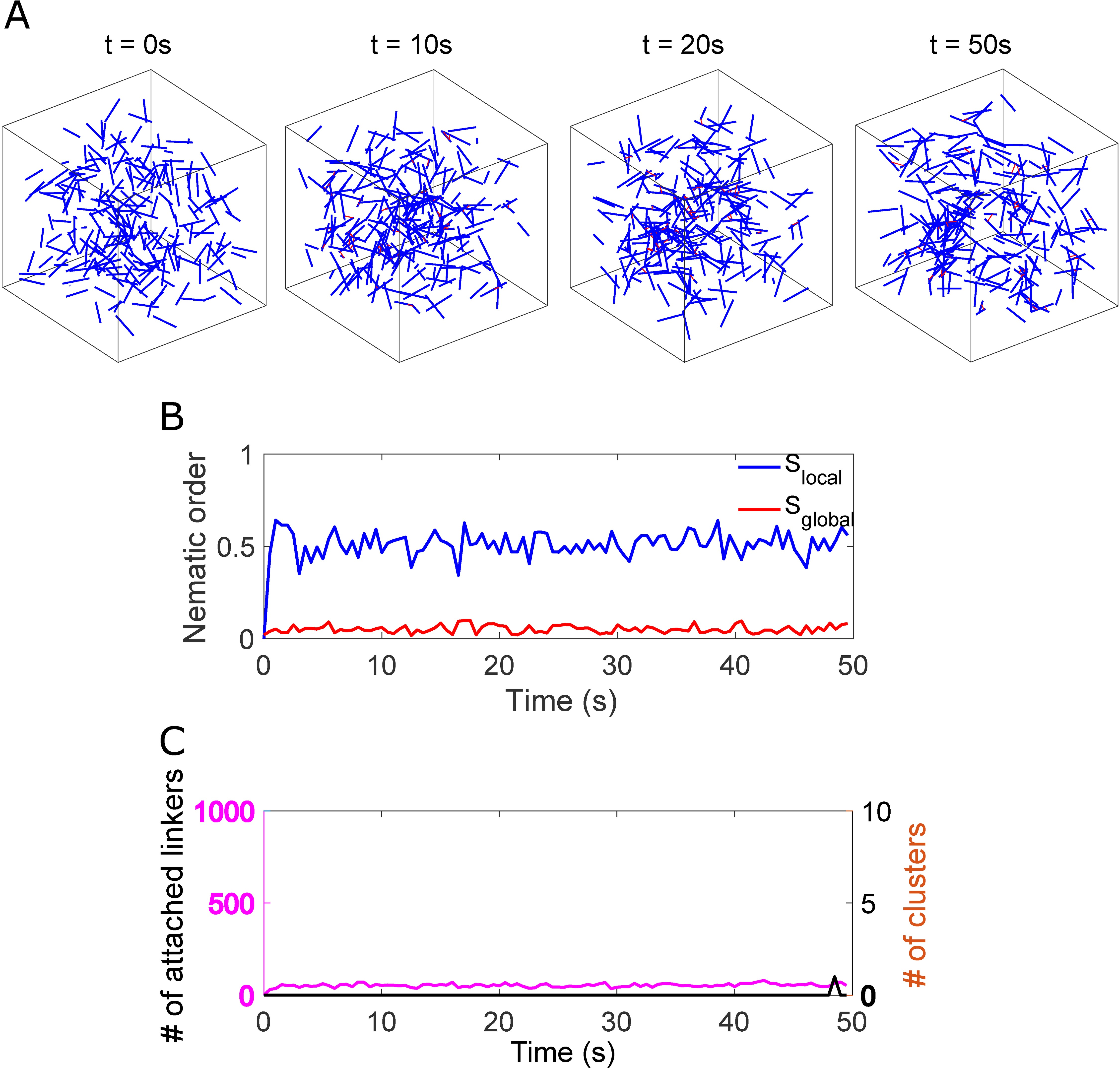

Supplement: S1 Fig — (A) Snapshots of an actin network with 81nm-long filaments in a 500nm-wide cubic box. The crosslinking rate is kf = 0.1s−1. Each filament is represented by a blue line and each crosslink by a red line. (B) Evolution of the local (blue) and global (red) nematic order parameter of the actin network over the course of the simulation shown in (A). (C) Evolution of the number of attached crosslinkers (magenta, left axis) and the number of clusters (black, right axis) for the simulation in (A). (TIF) [file pcbi.1006150.s001.tif]

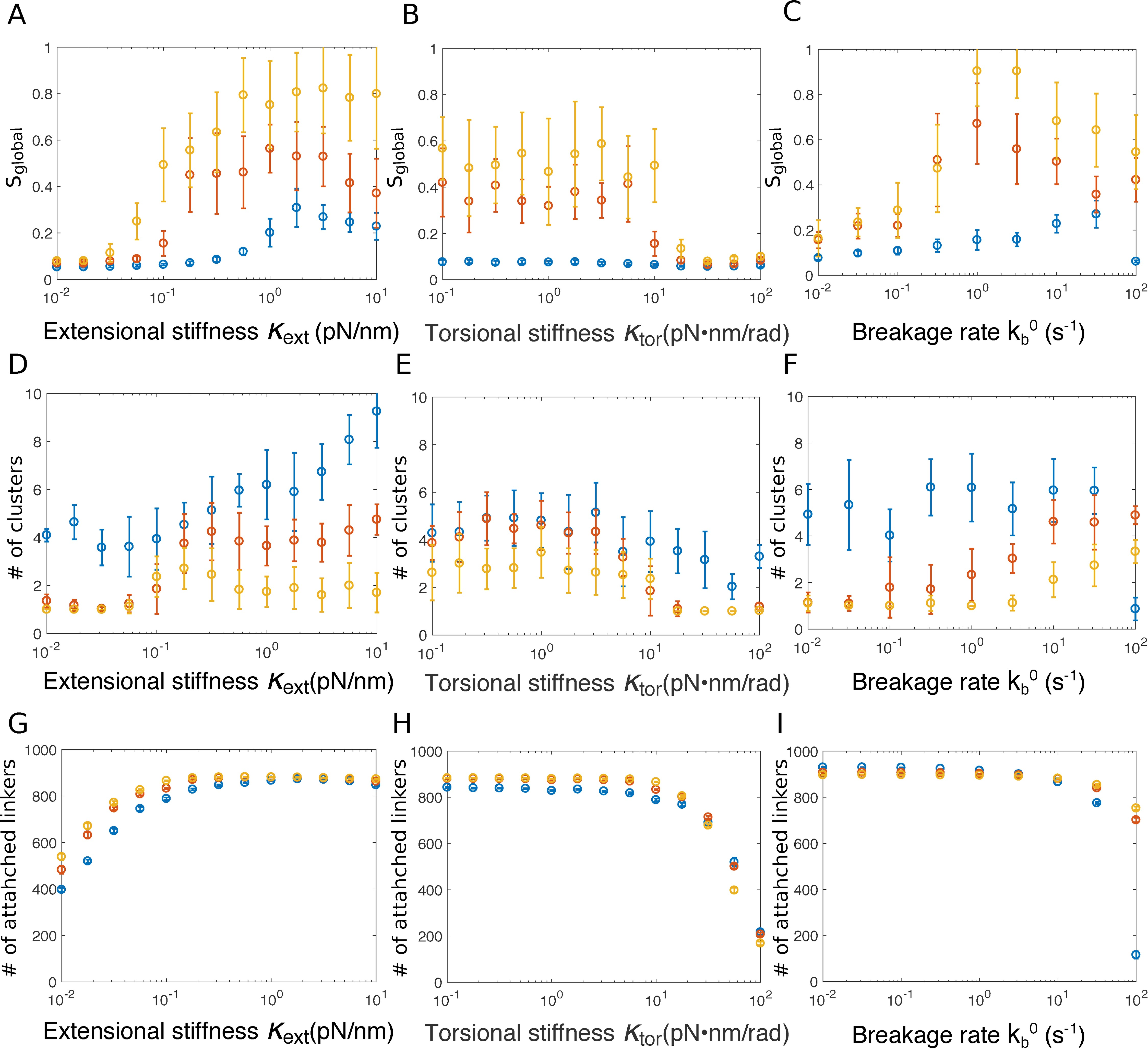

Supplement: S2 Fig — (A-C) Global nematic order parameter as a function of the extensional stiffness κext (A), the torsional stiffness κtor (B), and the linker breakage rate kb0 (C). (D-F) Number of clusters as a function of the same set of parameters. (G-I) Number of attached linkers as a function of the same set of parameters. Simulations are performed for filaments of various lengths: 81nm (blue), 135nm (red) and 189nm (orange). For each simulation, the means of the metrics were calculated from the data between 40s to 50s and the error bars indicate standard deviation over 10 simulations. (TIF) [file pcbi.1006150.s002.tif]

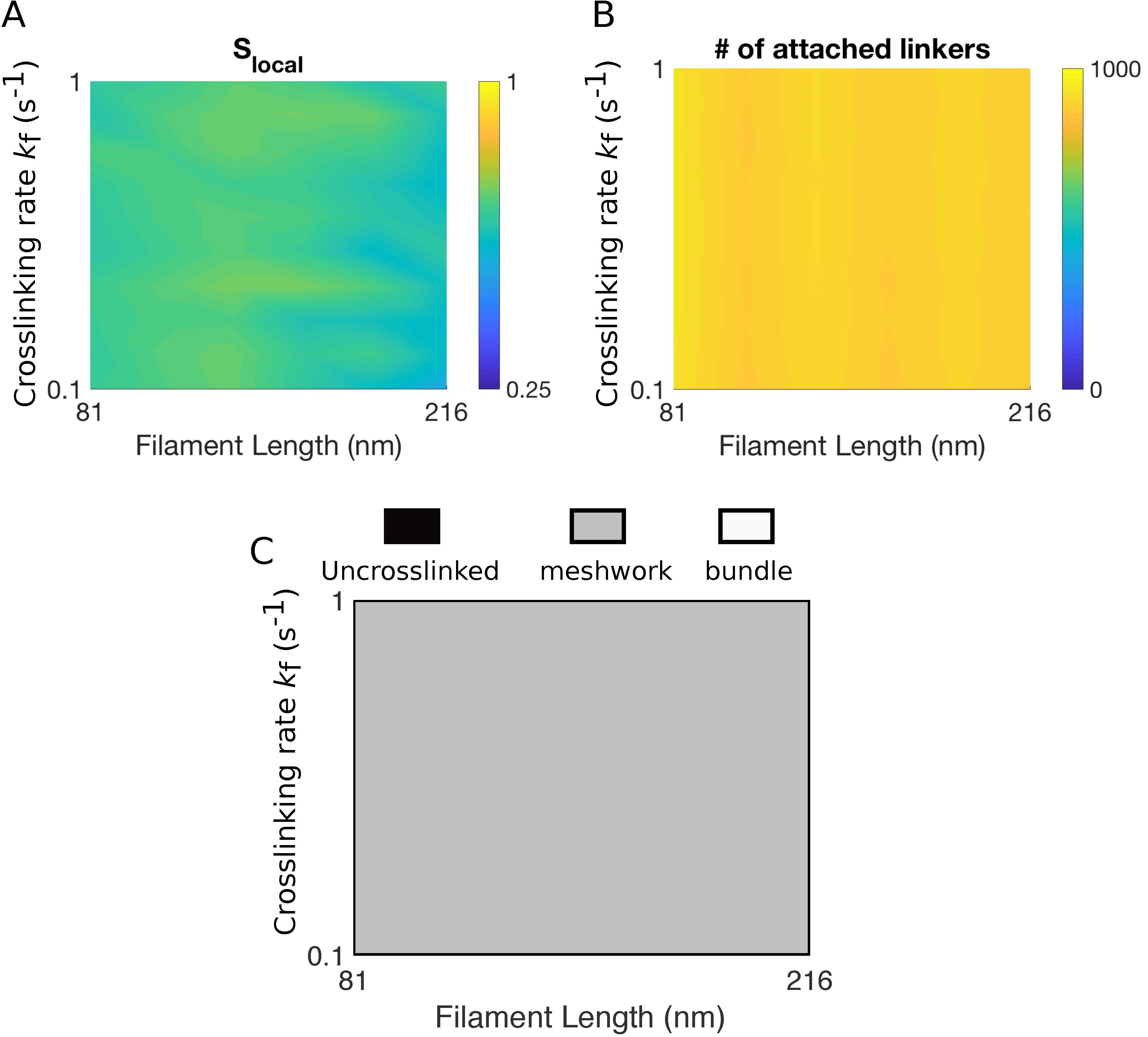

Supplement: S3 Fig — (A) Local nematic order parameter Slocal as a function of kf and L. (B) Number of attached crosslinkers Nattach as a function of kf and L. (C) Classification of actin network organizations as a function of kf and L. Meshworks occupy the entire parameter space. The criteria delimiting each possible organization are the same as in Fig 4E and 4G, i.e. Slocal = 0.75 and Nattach = 300. (TIF) [file pcbi.1006150.s003.tif]

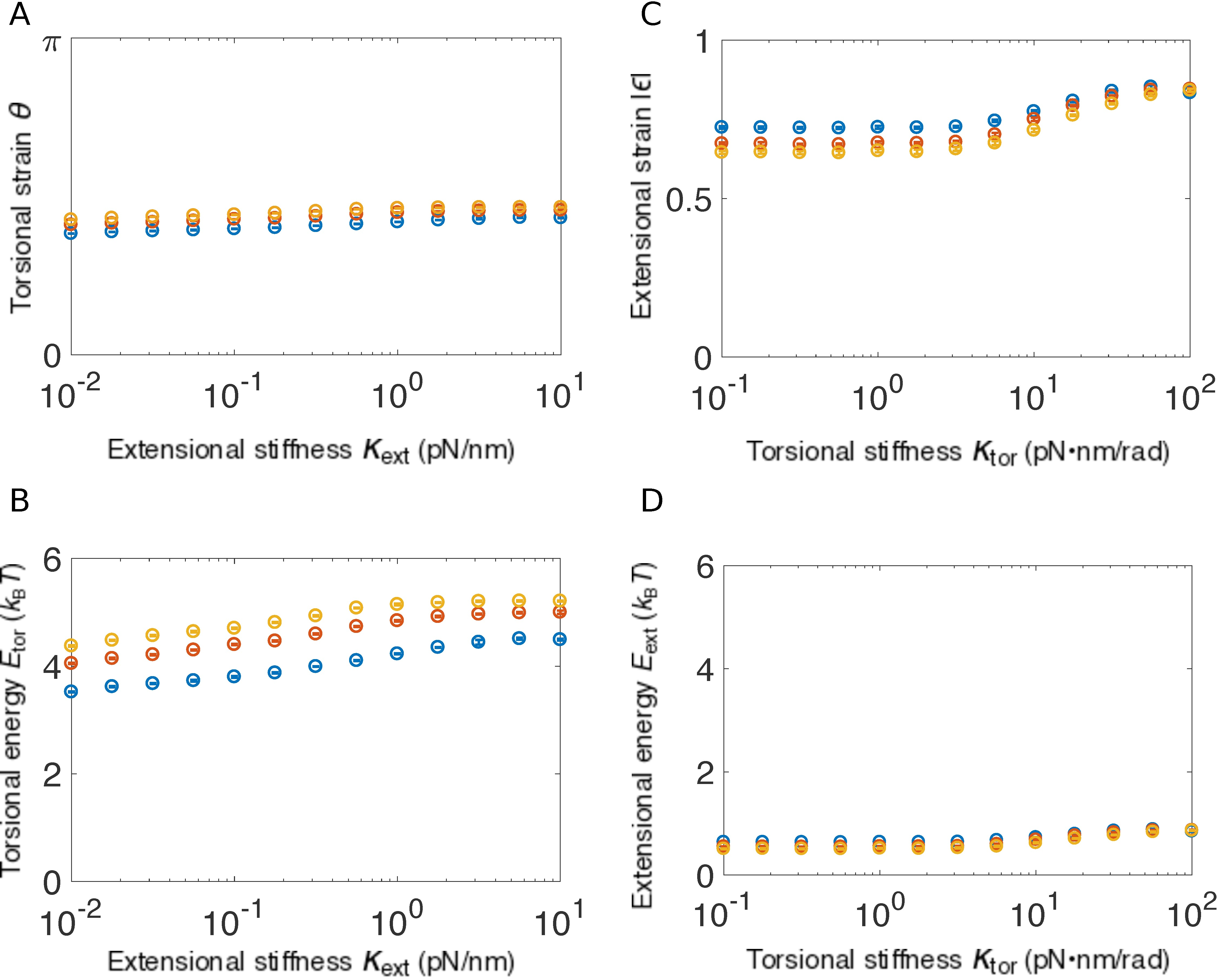

Supplement: S4 Fig — (A, B) Average torsional strain θ (A) and torsional energy (B) as a function of the extensional stiffness κext. (C, D) Average absolute value of the extensional strain |ϵ| (C) and extensional energy (D) as a function of the torsional stiffness κtor. Simulations were performed for filaments of various lengths: 81nm (blue), 135nm (red), and 189nm (orange). For each simulation, the means of the metrics were calculated from the data between 40s to 50s and the error bars indicate standard deviation over 10 simulations. (TIF) [file pcbi.1006150.s004.tif]

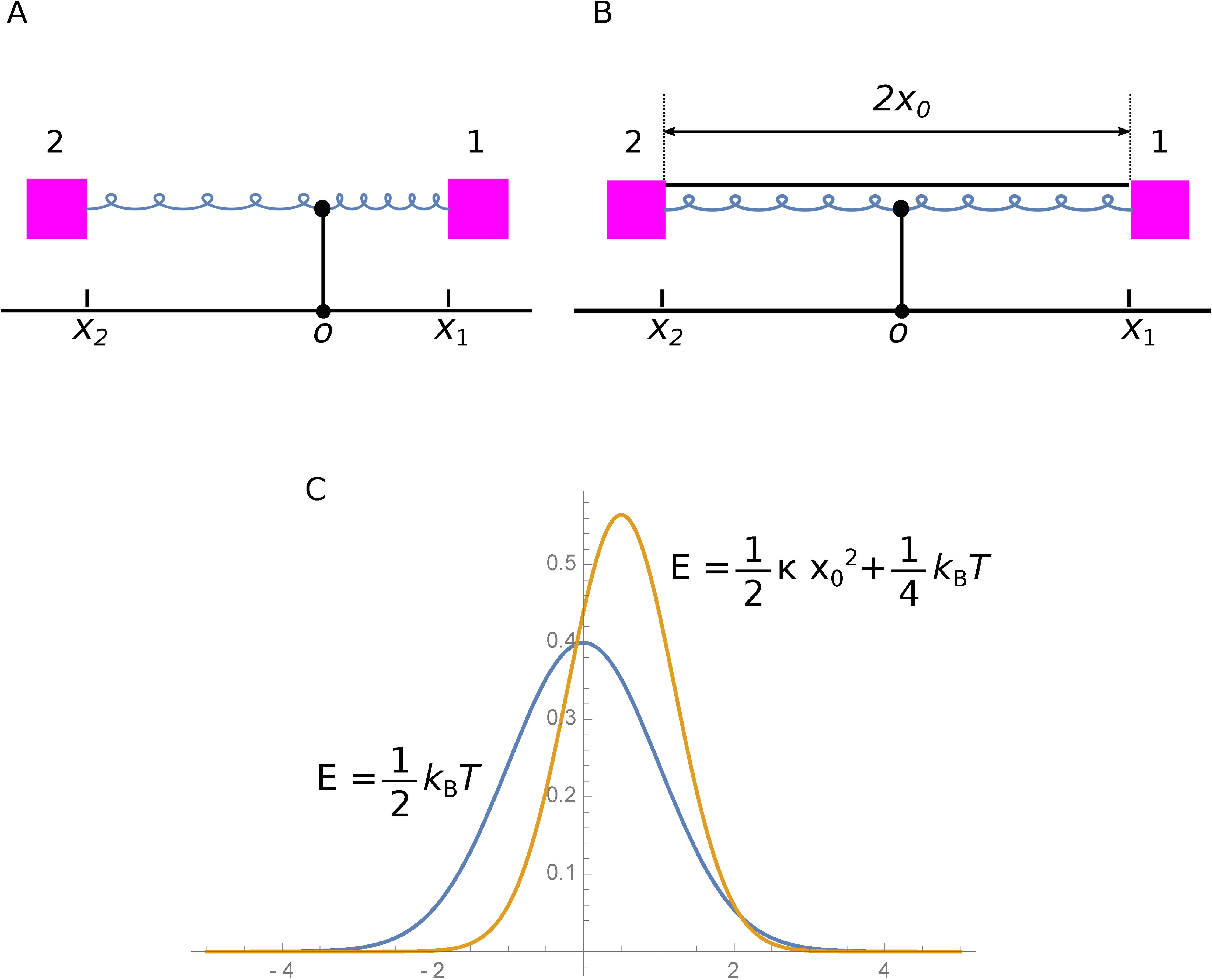

Supplement: S5 Fig — The black dot on the spring is constrained to stay at position 0. (A) The movements of the two particles are independent. (B) The movements of the two particles are strongly coupled (by the black bar) and constrained such that x1 − x2 = 2x0. (C) The position distribution of the particle 1 is shown in the decoupled case (blue) and in the coupled case (orange). (TIF) [file pcbi.1006150.s005.tif]

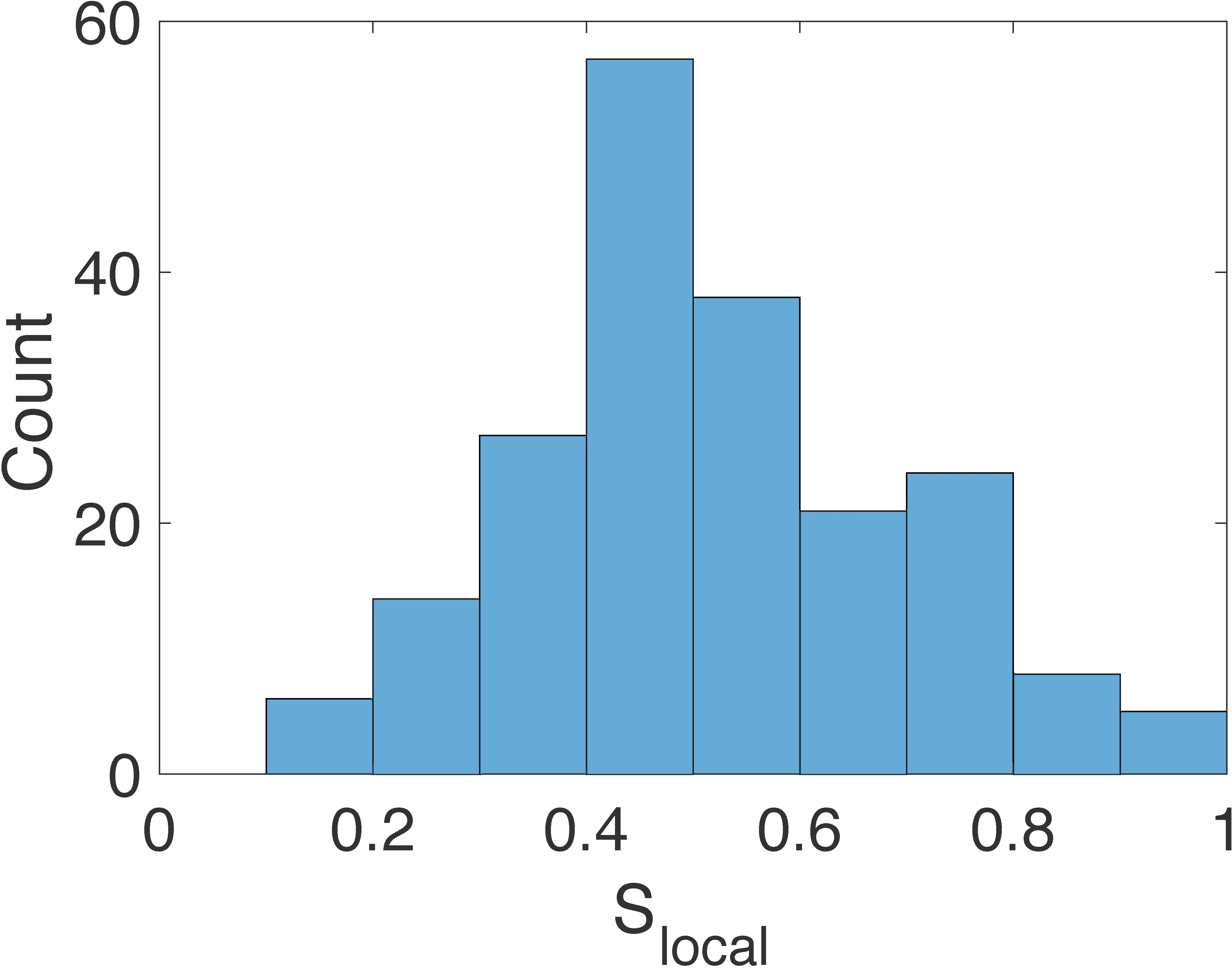

Supplement: S6 Fig — This distribution shows that randomly oriented vectors have a nematic order parameter centered around 0.4, rather than 0. The distribution was drawn from 200 simulations. (TIF) [file pcbi.1006150.s006.tif]

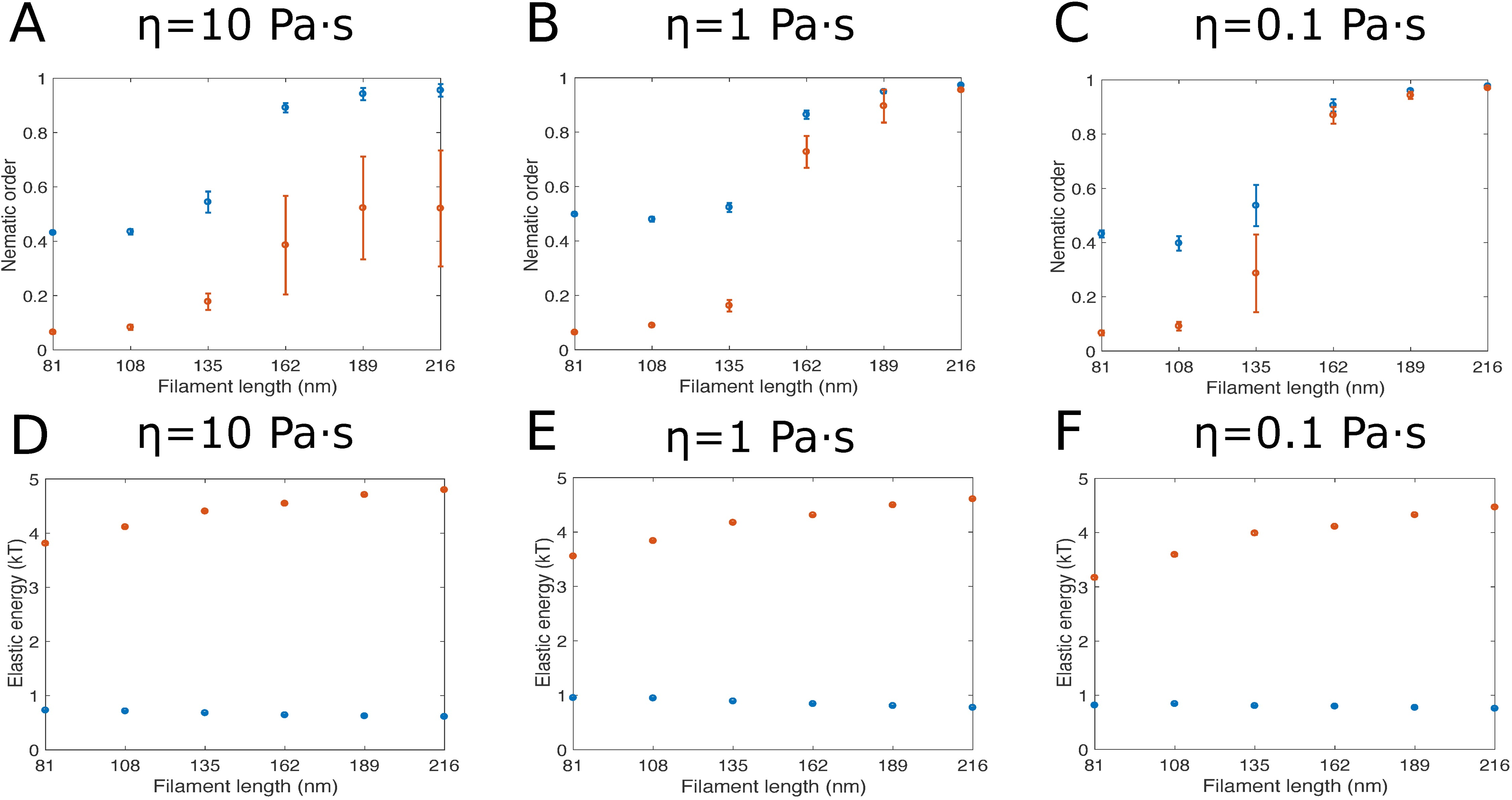

Supplement: S7 Fig — (A-C) Nematic order parameter as a function of filament length at various viscosities as indicated above the panel. Global and local nematic orders are shown in red and blue, respectively. (D-F) Elastic energy as a function of filament length at various viscosities as indicated above the panel. Extensional and torsional energies are shown in blue and red, respectively. The total simulation time is 10s for η = 0.1Pa ⋅ s, and is 50s for η = 1 − 10Pa ⋅ s. For each simulation, the means of the metrics were calculated from the data between 40s to 50s (for η = 1 − 10Pa ⋅ s) or between 8s and 10s (for η = 0.1Pa ⋅ s), and the error bars indicate standard deviation over 10 simulations. (TIF) [file pcbi.1006150.s007.tif]

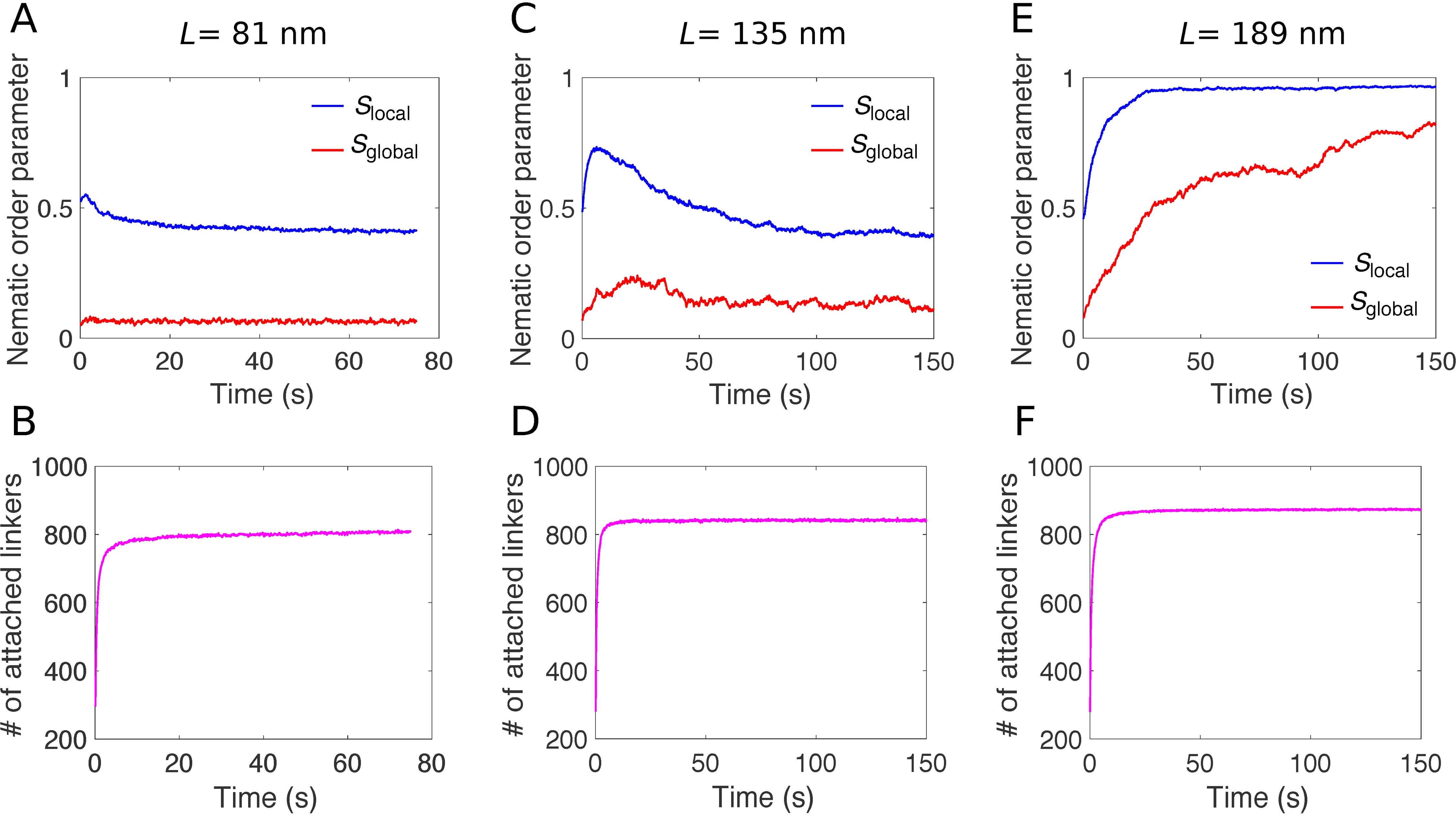

Supplement: S8 Fig — (A, C, E) Temporal evolution of local (blue) and global (red) nematic order parameter for filaments of length 81nm (A), 135nm (C) and 189nm (E). (B, D, F) Temporal evolution of number of attached crosslinkers for filaments of length 81nm (B), 135nm (D) and 189nm (F). All the metrics are averaged over 20 simulations for total 75s (A, B) or 150s (C-F). (TIF) [file pcbi.1006150.s008.tif]
